# Leveraging Advanced AI Frameworks for Dual PPAR α/γ Agonist Discovery in Alzheimer’s Disease

**DOI:** 10.1021/acschemneuro.6c00148

**Published:** 2026-07-16

**Authors:** Navaneet Chaturvedi, Vaibhav Mishra, Shafiul Haque, Sabiha Khatoon, Kamal Rawal, Vijay Kumar

**Affiliations:** † Amity Institute of Biotechnology, 77282 Amity University, Noida, Uttar Pradesh 201313, India; ‡ School of Medicine, Department of Neurology, University of Missouri, Columbia, Missouri 65211, United States; § Department of Nursing, College of Nursing and Health Sciences, 123285 Jazan University, Jazan 82911, Saudi Arabia; ∥ School of Medicine, Universidad Espiritu Santo, Samborondon 091952, Ecuador; ⊥ Department of Physiology and Biochemistry, 6186 University of Oklahoma Health Sciences Center, Oklahoma City, Oklahoma 73104, United States

**Keywords:** Alzheimer’s disease, PPAR, artificial intelligence, drug discovery, generative AI

## Abstract

The dual activating potential of peroxisome proliferator-activated receptors (PPAR) α and γ offers a promising way to address metabolic issues, neuroinflammation, and protein balance in the development of Alzheimer’s disease (AD). The current study intends to underscore the emerging importance of artificial intelligence (AI) in the accelerated discovery and optimization of dual PPAR α/γ modulators based on machine learning (ML), deep learning (DL), and generative modeling. The role of autonomous and agentic-AI systems in hypothesis generation, lead optimization, and closed-loop screening processes is highlighted. In addition, AI-enabled digital twin frameworks are emerging as powerful tools to integrate multiomics, neuroimaging, and clinical data for virtual modeling of disease progression and therapeutic response. Furthermore, the importance of explainable AI (XAI) in improving the interpretability and mechanistic insights of drug-receptor interaction models is underscored. Taken together, this review integrates recent studies illustrating the utility of AI-optimized dual PPAR α/γ agonists in enhancing lead selection, dose–response prediction, and therapeutic outcome prediction, thus bridging pharmacological computation and neuroimmune targeting for next-generation precision medicine approaches in AD therapy.

## Graphical abstract

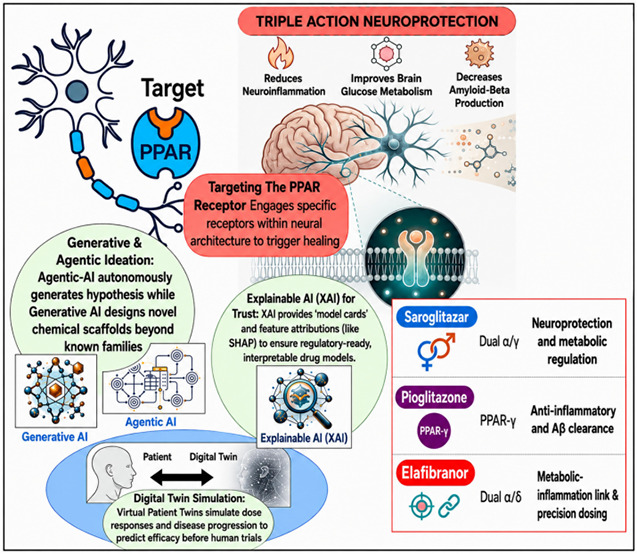

## 1 Introduction

Alzheimer’s disease (AD) is a leading cause of dementia and remains the most prevalent, causing almost 60–70% of dementia cases globally.^1^ AD has been described as a disorder characterized centrally by Aβ plaque formation, neurofibrillary tangles, chronic neuroinflammation, oxidative stress, and synaptic dysfunction, causing cognitive deterioration over time. Despite decades of research on AD, current treatments are symptomatic, placing AD as a major unmet medical need. Recent studies are now revealing AD as a metabolic dysfunction disorder, where lipid metabolism, mitochondrial, and insulin signaling pathways play a crucial part in Aβ and inflammatory signaling.^2^ In this context, activating dual PPAR, encompassing PPAR-α and PPAR-γ, has emerged as an exciting therapeutic strategy because of its capacity to regulate metabolic homeostasis and neuroinflammation.^3^ PPARα supports fatty acid oxidation and promotes Aβ clearance in glial cells, while PPARγ enhances insulin sensitivity and suppresses NF-κB–driven inflammation, together addressing key pathological axes of AD.^4–7^ Consistent with this mechanistic rationale, preclinical studies show that activation of either receptor can reduce amyloid burden and partially restore cognitive function in AD models.^8,9^

A structured literature survey was conducted using PubMed, Scopus, and Web of Science databases covering studies published up to 2025. Search terms included combinations of “Alzheimer’s disease,” “PPARα,” “PPARγ,” “dual PPAR agonist,” “artificial intelligence,” “machine learning,” “deep learning,” and “drug discovery.” Studies were included if they reported AI-driven methodologies, PPAR-targeted therapeutic approaches, or multiomics integration relevant to AD. Studies not directly related to neurodegeneration, lacking AI-based approaches, or without sufficient methodological detail were excluded. Notably, due to substantial heterogeneity in study design and outcome measures, including cognitive assessments, neuroimaging biomarkers, and omics-based surrogate end points, a formal meta-analysis was not performed. Therefore, findings are presented as a qualitative synthesis intended to highlight emerging trends and conceptual advances.

In parallel, explainable artificial intelligence (XAI) tools will facilitate the interpretation of models representing drug-target interaction phenomena, while federated learning tools support the analysis of multi-institutional AD data sets while protecting patient privacy. The implementation of digital twin paradigms, defined as computational models of specific molecular, cellular, and clinical states, unifies the frameworks for simulating the dynamics of the PPAR α/γ pathway, predicting clinical outcomes, and facilitating the implementation strategies of precision medicine approaches in the treatment of AD. These informatics-based technologies create a foundation for the next generation of AI-based discovery, optimization, and lead generation of dual PPAR α/γ modulators, which exhibit expanded translational potential for the treatment of AD. To illustrate the high speed at which AI technologies, research paradigms, and the biology underlying AD, including PPAR, have evolved, we employed a bibliometric co-occurrence analysis, where Figure 1 illustrates co-occurrence patterns and thematic associations between artificial intelligence and AD research domains. These visualizations reflect patterns of keyword comention and should be interpreted as exploratory representations rather than statistically validated evidence of domain convergence.

**Figure 1:**
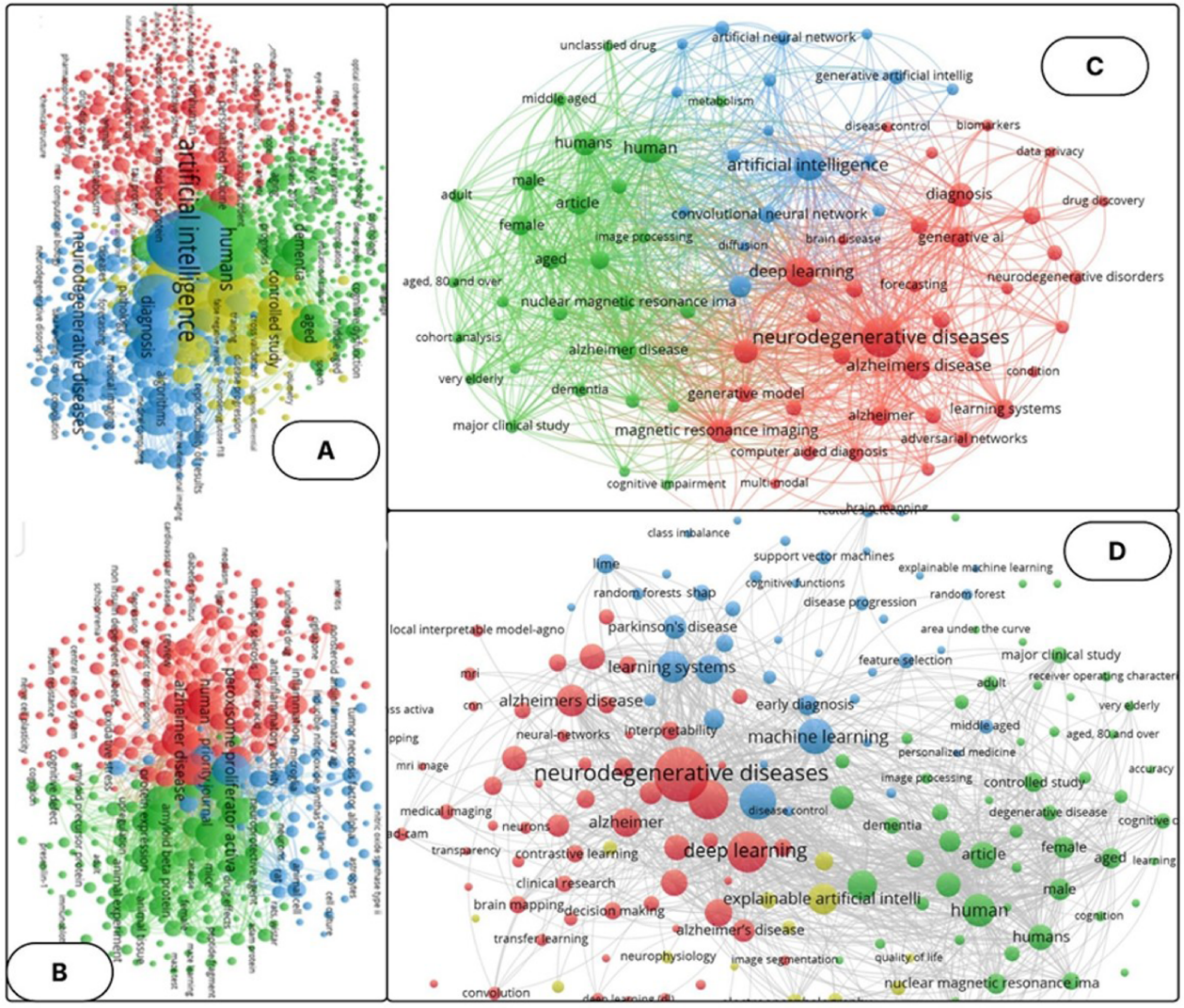
Bibliometric keyword co-occurrence network visualizations generated using VOSviewer (https://www.vosviewer.com/) illustrating thematic relationships between artificial intelligence (AI) and Alzheimer's disease (AD) research. (**A**) AI–AD keyword network; (**B**) PPAR- and AD-related research themes; (**C**) Generative AI-related keyword associations; and (**D**) Explainable AI (XAI) and machine learning-related keyword associations. Node size reflects keyword occurrence frequency, and links represent keyword co-occurrence. These networks provide qualitative representations of thematic associations and do not constitute statistically validated measures of research convergence or integration.

In this review, we critically synthesize current evidence supporting dual PPAR α/γ targeting as a multifactor therapeutic strategy for AD and examine how recent advances in AI are accelerating its discovery and optimization. We integrate development in ML, DL, generative modeling, and emerging agentic AI frameworks that enable autonomous hypothesis generation, lead optimization, and closed loop screening of PPAR α/γ modulators. Particular emphasis has been placed on the role of XAI in enhancing interpretability, mechanistic insight, and confidence in drug receptor interaction models, as well as federated and multi-institutional data learning approaches for privacy-preserving analysis of AD data sets. Thereby, the review highlighted representative case studies illustrating AI-guided lead selection, dose response prediction, patient stratification, and therapeutic outcome modeling.

The application of AI workflows may allow an interface between molecular discovery and patient-specific precision medicine for dual PPARα/γ agonists in AD (Figure 2). Figure 2 graphically depicts an AI-based drug-discovery process, which includes the screening of virtual compound libraries by trained and validated models to identify promising dual PPARα/γ agonists. The model includes processes for model tuning and cross-validation, encompassing linkages from predictive computations to neuroprotection end points like decreased neuroinflammation, enhanced glucose metabolism in the brain, and inhibition of amyloid-β production. Using such a model, applications of AI may enable the integration of multiomics data sets, high-throughput screen data, and structural data to assign new dual PPARα/γ agonists into different categories, predict blood-brain barrier (BBB) permeability, and enhance pharmacokinetic and toxicity properties.^10,11^ Moreover, applying AI may enable precision medicine strategies through patient stratification, prediction, and prescribing adaptive dosing strategies.^12,13^

**Figure 2:**
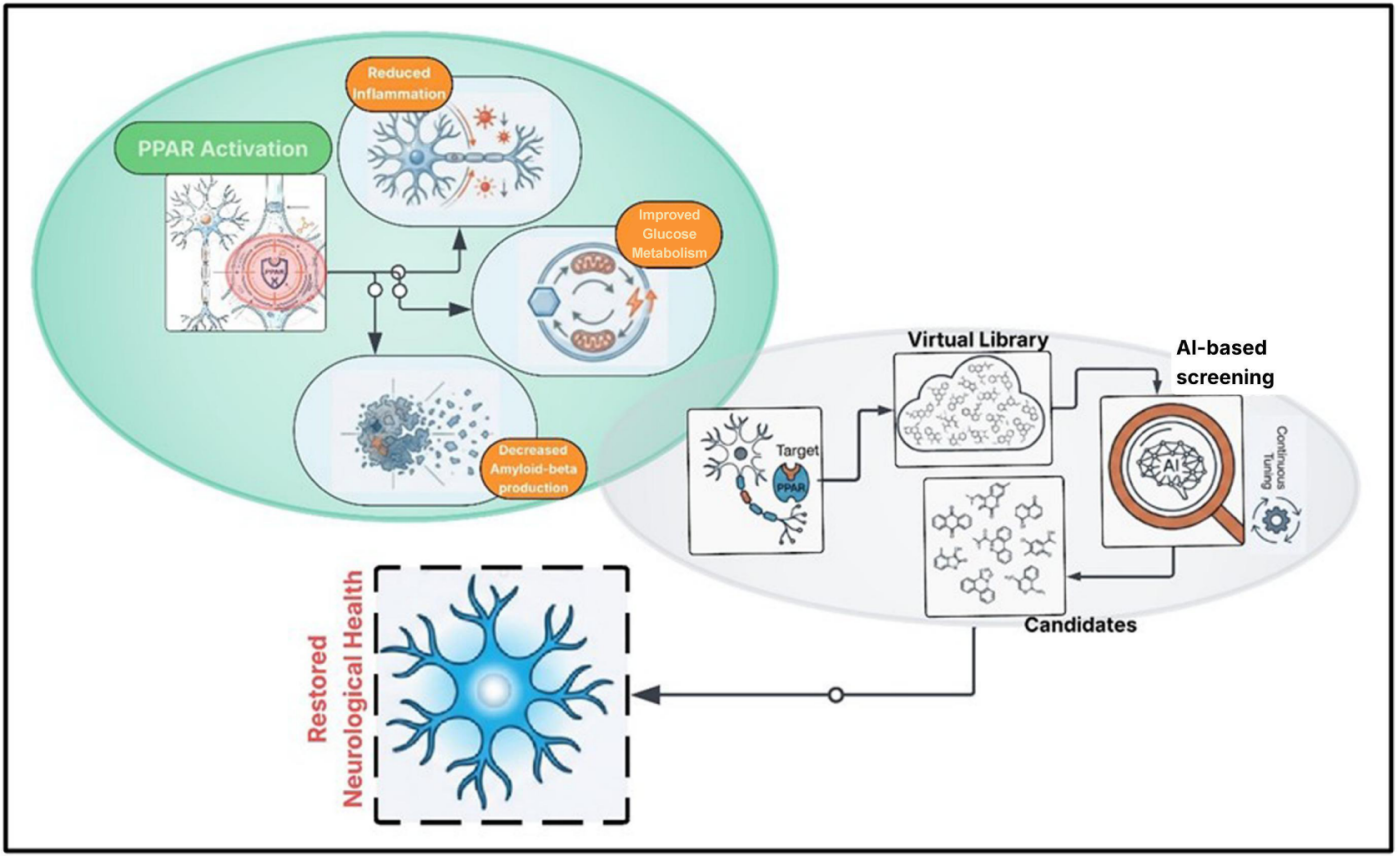
AI-driven virtual screening workflow in which an optimized machine-learning model screens large chemical libraries to prioritize neuroactive candidates and link computational predictions to therapeutic effects such as reduced neuroinflammation, improved brain glucose metabolism, and decreased amyloid-β production.

## 2 AI Approaches for Identifying Dual PPARα/γ Agonists

For clarity, studies discussed in this review are categorized based on receptor specificity into PPARα-only, PPARγ-only, dual PPARα/γ, and other PPAR isoform-targeting agents (e.g., PPARα/δ). Mechanistic interpretations regarding dual PPARα/γ agonism are restricted to studies directly investigating dual receptor modulation, whereas findings from single-receptor or alternative isoform agonists are discussed primarily to illustrate the application of AI-driven methodologies.

Dual activation of PPARα/γ targets multiple pathogenic mechanisms in AD, including metabolic dysfunction, neuroinflammation, and impaired lipid handling,.^8,14^ In addition, advanced AI models, spanning ML and DL have transformed the discovery and optimization of PPAR agonists, that rapidly develop new compounds with high therapeutic potential. Even though, ML models have successfully predicted PPARγ agonists as AD therapeutics.^7^ Beyond drug discovery, AI tools also clarify disease mechanisms. For example, metabolomics studies using multilayer perceptron and extreme-gradient boosting algorithms revealed metabolic signatures relevant to AD and PPAR signaling. Hence, integrated in silico workflows likewise identify natural products with multitarget anti-AD activity.^15^ Additionally, PPAR agonists, including pioglitazone for PPARγ,^16^ fenofibrate for PPARα,^17^ and the dual agonist saroglitazar^18^ which have shown cognitive and biomarker benefits in preclinical and early clinical settings.

### 2.1 Rationale for Dual PPARα/γ Agonism in AD

Insulin resistance and impaired cerebral glucose metabolism are key drivers of AD pathology. Whereas, dual PPARα/γ agonists address these key issues simultaneously, reduce pro-inflammatory cytokines and enhance anti-inflammatory mediators in patient-derived microglia, modulating the neuroimmune microenvironment.^19^ While dual PPARα/γ activation is proposed to enable coordinated regulation of metabolic and inflammatory pathways, the absence of quantitative systems-level modeling and comparative analysis limits the ability to determine whether such effects are additive, synergistic, or antagonistic. Although, PPARγ activation improves insulin sensitivity and glucose utilization (Figure 3), while PPARα stimulation augments lipid metabolism and mitochondrial function, restoring cerebral metabolic balance.^20,21^ Further, ML analyses of transcriptomic and proteomic data sets further reveal downstream targets and regulatory pathways affected by PPAR activation.^22^ Although DL-based virtual screening has uncovered novel neuroprotective compounds,^23^ while graph neural networks (GNNs) predict PPARα/γ binding affinities and having potential to accelerate the search for candidates with balanced dual activity.^24^

**Figure 3:**
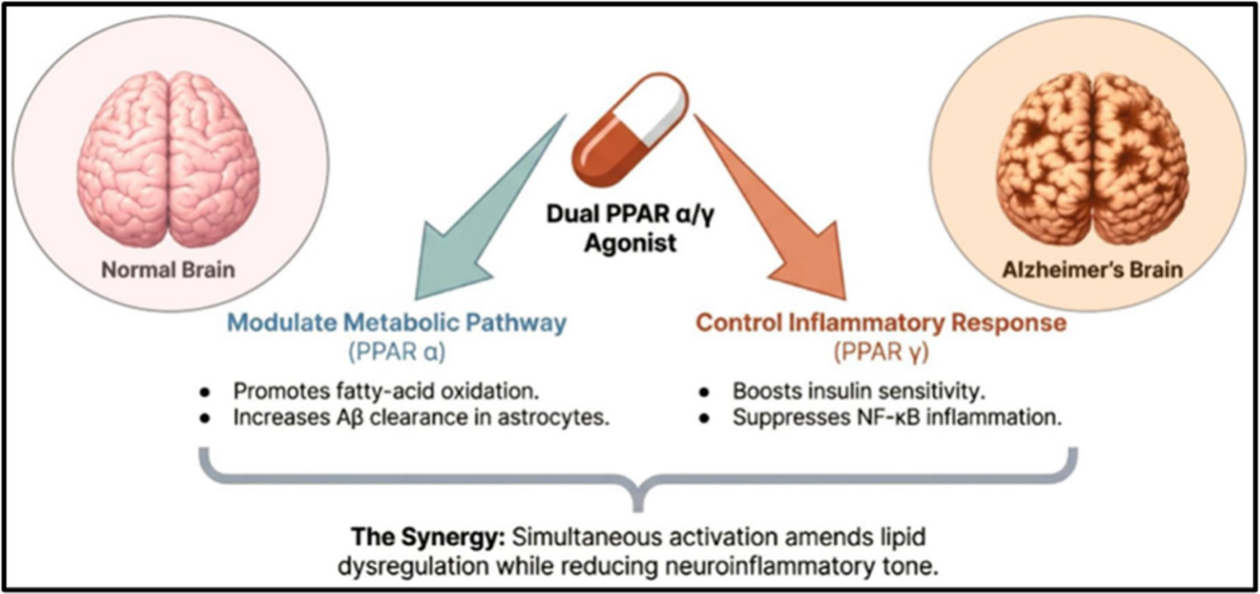
Conceptual schematic illustrating the proposed mechanisms of dual PPARα/γ activation in modulating metabolic and inflammatory pathways. The figure highlights potential complementary roles of receptor activation; however, it does not represent a quantitative systems model and does not include validated dose–response relationships, receptor occupancy, or comparative performance relative to single-receptor activation.

### 2.2 ML for Predicting PPARα Agonist Efficacy in AD

ML methods from classical algorithms to advanced DL models like GNNs, RF, NLP, and generative (Table 1), which analyze large genomics, proteomics, metabolomics, and clinical data sets to identify predictive biomarkers.^25^ In addition, random forest (RF) model integrating genetic polymorphisms, blood biomarkers, and neuroimaging features predicted cognitive improvement after fenofibrate (PPARα agonist) treatment with 78% accuracy.^26^ Whereas as discussed above GNNs applied to multiomics and protein–protein interaction networks achieved an AUC-ROC of 0.85 when modeling the interplay between PPARα activation and AD-related pathways.^27,28^ Although generative models, including variational autoencoders (VAEs) and generative adversarial networks (GANs), design novel PPARα agonists with improved blood-brain barrier penetration and target engagement.^29^ Further natural-language processing (NLP) applied to >50,000 electronic health records found that long-term PPARα agonist use was associated with a 15% reduction in cognitive decline, with greater benefits in specific genetic subgroups.^30,31^

**Table 1:** Comparison of ML Methods in Alzheimer’s Drug Design, Detailing Data Sources, Goals, and Outcomes for Random Forest (RF), Graph Neural Networks (GNNs), and Generative Models

Characteristics	Random Forest	GNNs	Generative Models
Data Used	Genetic polymorphisms, blood biomarkers, neuro-imaging features	Multiomics data, protein–protein interaction network	Molecular docking simulations
Goal	Predict cognitive improvement	Analyze interplay between PPARα activation and AD0related pathways	Design novel PPARα agonist with improved properties
Outcome	78% accuracy in prediction	0.85 AUC-ROC in predicting efficacy	Enhanced neuroprotective properties

### 2.3 Deep Learning (DL) Analysis of PPAR Expression in AD Brain Tissue

DL provides powerful tools to dissect PPAR expression patterns in AD brains, illuminating disease mechanisms and therapeutic opportunities. PPARα, PPARβ/δ, and PPARγ regulate metabolism, inflammation, and cell differentiation, and their regional expression correlates with insulin resistance, lipid dysregulation, and neuroinflammation.^32^ Although modern DL models like convolutional and recurrent neural networks (CNNs & RNNs) excel at image and time-series analysis and have revealed complex PPAR expression patterns in transcriptomic and proteomic data sets.^33,34^ CNNs have identified spatial PPARγ expression relative to Aβ plaques and neurofibrillary tangles, while GNNs integrating single-cell and spatial transcriptomics revealed interactions between PPARα-expressing microglia and neighboring neurons.^35^ Although DL offers unprecedented analytical power, challenges include interpretability and overfitting; experimental validation remains essential.^36^ Figure 4 illustrates representative AI applications, that include CNN-based expression for combination therapy and summarizes major DL case studies.

**Figure 4:**
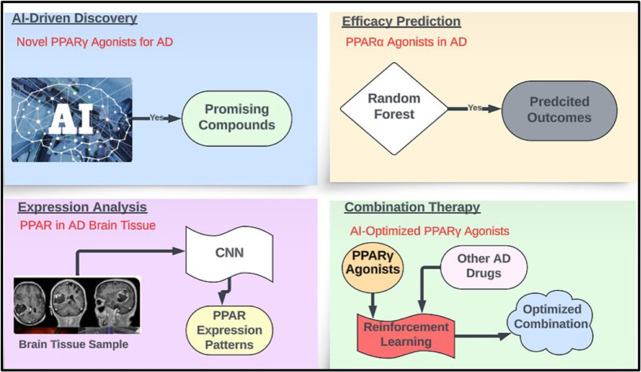
Illustration of AI applications in PPAR-targeted Alzheimer’s therapy: AI-based discovery of novel PPARγ agonists, prediction of PPARα agonist efficacy using Random Forest, CNN-based expression analysis in brain tissue, and reinforcement learning for optimized combination therapy.

### 2.4 Generative AI-Driven Design of Dual PPARα/γ Agonists for AD

Generative AI represents a natural evolution of AI-driven drug discovery, particularly suited to complex disorders such as AD that require multitarget pharmacological strategies. Generative-AI approaches actively design new chemical entities by learning the structural and physicochemical rules that govern molecular bioactivity, in contrast to traditional machine learning models that mainly rank or classify existing compounds.^37^ This ability is particularly useful for the discovery of dual PPAR α/γ agonists, as it is still difficult to achieve a balanced modulation of both receptors while preserving drug-likeness for the central nervous system (Figure 5).

**Figure 5:**
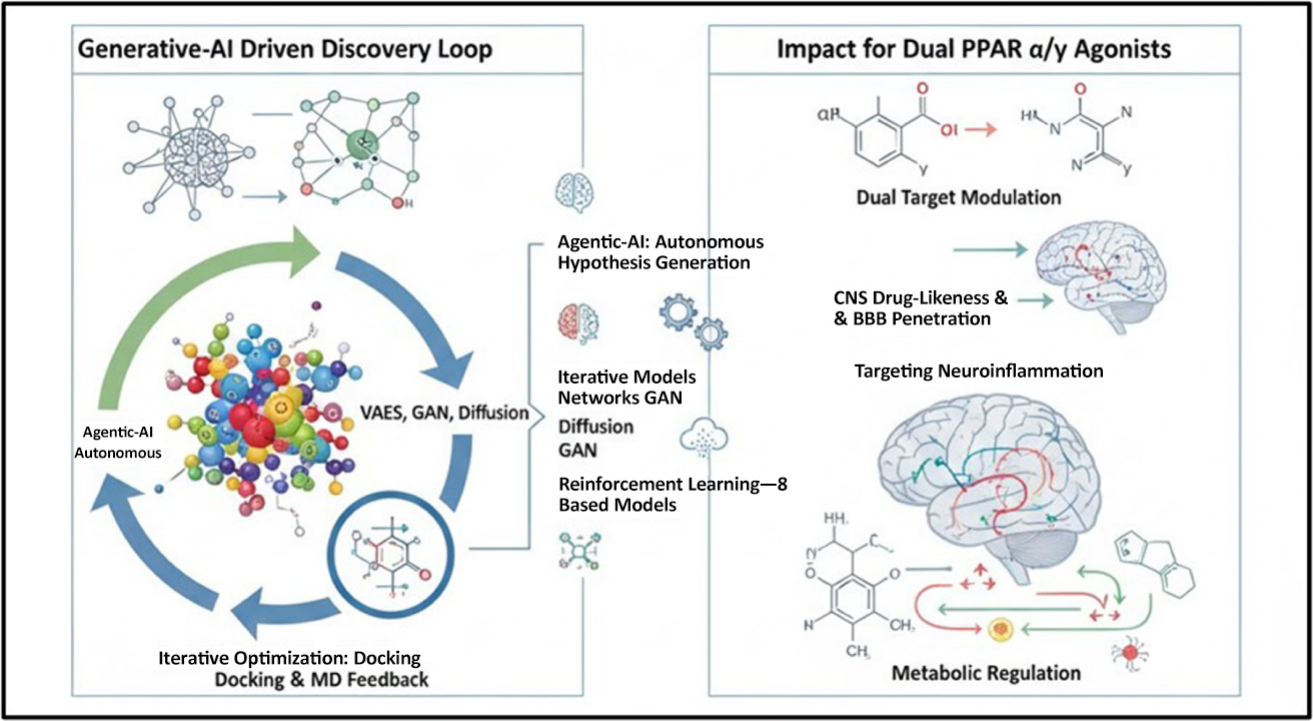
Generative AI-driven discovery loop for dual PPARα/γ agonists, integrating agentic and generative models with iterative docking and molecular dynamics feedback to optimize dual-target activity and CNS drug-likeness for AD.

Many classes of generative-AI models have been applied to address these challenges, including variational autoencoders, generative adversarial networks, diffusion-based models, and reinforcement learning-guided molecular generators (Table 2). These structures encourage scaffold innovation outside of recognized PPAR ligand families and facilitate effective exploration of vast latent chemical spaces. Through the integration of structure-based constraints, pharmacophore features, and polypharmacological objectives, generative models are able to suggest candidate molecules that can engage PPAR α and PPAR γ at the same time while minimizing predicted toxicity and off-target interactions.^38^

**Table 2:** Representative Generative-AI Models Applied to Dual PPARα/γ–Oriented Drug Discovery

Generative-AI Model Class	Representative Architectures	Application in Dual PPAR α/γ Discovery	Key Advantage for AD Drug Design
Variational Autoencoders (VAEs)	Molecular VAEs, Junction Tree VAEs	Latent-space generation of novel PPAR scaffolds with tunable α/γ selectivity	Smooth optimization of multiobjective properties (affinity, BBB penetration)
Generative Adversarial Networks (GANs)	MolGAN, GraphGAN	De novo synthesis of chemically valid and diverse dual-target ligands	Enhanced scaffold diversity beyond known PPAR chemotypes
Diffusion Models	Molecular diffusion probabilistic models	Progressive refinement of ligand structures toward optimal dual-binding profiles	High chemical validity and fine-grained structural control
Reinforcement Learning–Based Models	Policy-gradient and reward-driven generators	Optimization of ligands using dual PPAR activation and ADMET-based reward functions	Direct alignment with therapeutic and safety objectives
Hybrid Generative Pipelines	Generative AI + Docking + MD feedback	Iterative lead optimization through closed-loop AI workflows	Accelerated convergence toward clinically relevant candidates

Additionally, iterative optimization loops, where model-generated compounds are improved using input from molecular docking, molecular dynamics simulations, and in silico ADMET predictions, are increasingly incorporating generative-AI workflows.^39^ These workflows become adaptive when combined with agentic-AI systems, enabling independent molecular scaffold redesign and prioritization based on changing performance metrics.^40,41^ In parallel, XAI contributes interpretability by highlighting molecular features that drive dual PPAR activation, thereby improving mechanistic confidence and translational readiness. Together, these advancements position generative AI as a crucial component of precision-guided discovery pipelines for next-generation dual PPAR α/γ agonists that target AD.

## 3 Data Resources and XAI

The AD Neuroimaging Initiative offers longitudinal neuroimaging, cerebrospinal fluid biomarkers, and cognitive assessments, providing surrogate end points and patient stratification capabilities.^42^ Community data sets play a crucial role in developing generalizable AI models. Multiomics resources for AD, such as the AMP-AD/AD Knowledge Portal, integrate harmonized genomic, transcriptomic, proteomic, metabolomic, and clinical data across large cohorts and experimental systems.^43^ In addition, proteomics and metabolomics repositories, such as PRIDE and the broader ProteomeXchange network, enable integration of mass-spectrometry data sets with transcriptomic profiles for pathway and target validation.^44^ These resources form a robust foundation for reproducible AI-based PPARα/γ discovery pipelines (Table 3). Robust AI-motivated discovery of dual PPARα/γ agonists requires careful attention to the data foundation and the explainability/trustworthiness of models used to prioritize compounds, predict brain penetration, and stratify patients. Table 3 outlines the essential elements of a complete AI pipeline capitalized in the discovery and optimization of dual PPARα/γ agonists for treatment in AD. The data were integrated from diverse sources like AMP-AD, ADNI, ROSMAP, ChEMBL, BindingDB, PDB, and PRIDE to allow coverage from multiomics and cheminformatics analyses. The need for reproducibility, explainability, and integrity is deep-rooted in each step followed by the AI pipeline. This begins from standardized quality control as well as batch corrections, which continues through the inclusion of other validation sets. The application of SHAP and Integrated Gradient analysis is recommended, alongside which uncertainty quantification is included. The crucial aspect of FAIR data is addressed through the employment of model cards. The AI pipeline’s application of federated learning methods is well-emphasized, which helps advance AI-enabled ethical and reproducible discovery related to PPAR-targeted therapeutics.

**Table 3:** Key Public Data Resources for AI-Motivated PPARα/γ Discovery, Including Data Types, Access, and Representative AD/PPAR Use Cases

Resource	Data Types	Access	Representative AD/PPAR Use Case
AMP-AD/AD Knowledge Portal	Genomics, transcriptomics, proteomics, metabolomics, clinical phenotypes	Public, controlled-access for some data sets	Train models linking PPAR pathway signatures to cognitive decline or imaging biomarkers
ADNI (AD Neuroimaging Initiative)	Longitudinal MRI/PET imaging, CSF biomarkers, cognitive scores, genomics	Public with data use agreement	Train BBB-penetration predictor from PET + plasma metabolomics
ROSMAP	Multiomics (RNA-seq, proteomics, metabolomics), histology, clinical data	Controlled-access via data use agreement	Validate PPARα/γ target signatures in postmortem brain tissues
ChEMBL	Bioactivity (IC_50_, *K*_d_, EC_50_), chemical structures, target annotations	Public	Supervised ML on PPAR ligand binding and potency
BindingDB	Measured binding affinities, assay data, molecular structures	Public	Curate PPARα/γ ligand affinities for predictive modeling
PubChem	Chemical structures, bioactivity summaries, ADMET metadata	Public	Retrieve chemical descriptors for molecular feature engineering
DrugBank	Approved drugs, clinical annotations, ADMET properties	Public	Annotate drug-likeness and safety profiles of candidate PPAR agonists
Protein Data Bank (PDB)	3D protein structures, ligand-bound complexes	Public	Structure-based docking and modeling of dual PPAR binding
PRIDE/ProteomeXchange	Mass spectrometry proteomics data sets	Public	Integrate proteomics signatures with transcriptomics for pathway validation

Next, we summarize key public resources, data types and preprocessing steps, integration strategies, and XAI techniques and uncertainty quantification methods that are essential for reproducible, defensible drug-discovery pipelines (Table 4, Figure 6). Further, ROSMAP delivers deeply phenotyped clinical and postmortem multiomics data widely used for discovery and validation.^45^ These resources enable the training of AI models that link PPAR pathway signatures with clinical decline or imaging biomarkers. Although bioactivity and assay data, such as IC_50_, *K*_d_, and EC_50_ from ChEMBL or BindingDB, must be harmonized in terms of units and assay types, with low-confidence records filtered. Whereas cheminformatics and bioactivity databases, including ChEMBL and BindingDB, provide curated ligand-target binding affinities and assay data that are essential for supervised ML on PPAR targets. Complementary chemical information, including molecular structures and ADMET annotations, is available from PubChem and DrugBank, while 3D receptor structures can be retrieved from the Protein Data Bank (PDB) for docking and structure-based modeling.^46^ Additionally, AI models rely on high-quality, molecular and chemical features, including SMILES, InChI strings, computed descriptors, and 3D coordinates for docking, require careful standardization of tautomers, stereochemistry, and protonation states.^47^ Clinical and imaging data require harmonization of cognitive scores, medication histories, and standardized image preprocessing pipelines.^48^

**Table 4:** A Uniform AI-Enabled Pipeline for Integrative AD Modeling and Drug Discovery, Outlining Sequential Steps from Multisource Data Integration and Quality Control to Explainable, Uncertainty-Aware, and Federated learning-Based Model Deployment under FAIR Compliance

Step	Pipeline Component	Description/Key Action
1	Data Inventory and Integration	Collect and link raw data from key sources (AMP-AD, ADNI, ROSMAP, ChEMBL, BindingDB, PDB, PRIDE) for multiomics, clinical, and cheminformatics integration.
2	Quality Control (QC) and Batch Correction	Apply standardized quality control, normalization, and batch correction using version-controlled analytical tools to ensure data consistency and reproducibility.
3	External Validation	Reserve independent validation cohorts to assess model generalizability and prevent overfitting.
4	Feature Attribution and Explainability	Compute and report feature importance using XAI (e.g., SHAP, Integrated Gradients) to interpret model predictions.
5	Uncertainty Quantification	Quantify model uncertainty and flag predictions with high uncertainty to improve decision reliability.
6	Model Documentation and FAIR Compliance	Generate a “model card” detailing architecture, performance, and limitations; deposit data sets following FAIR (Findable, Accessible, Interoperable, Reusable) principles.
7	Federated and Secure Learning	Implement federated learning and secure data aggregation protocols to enable multi-institutional collaboration without compromising data privacy.

**Figure 6:**
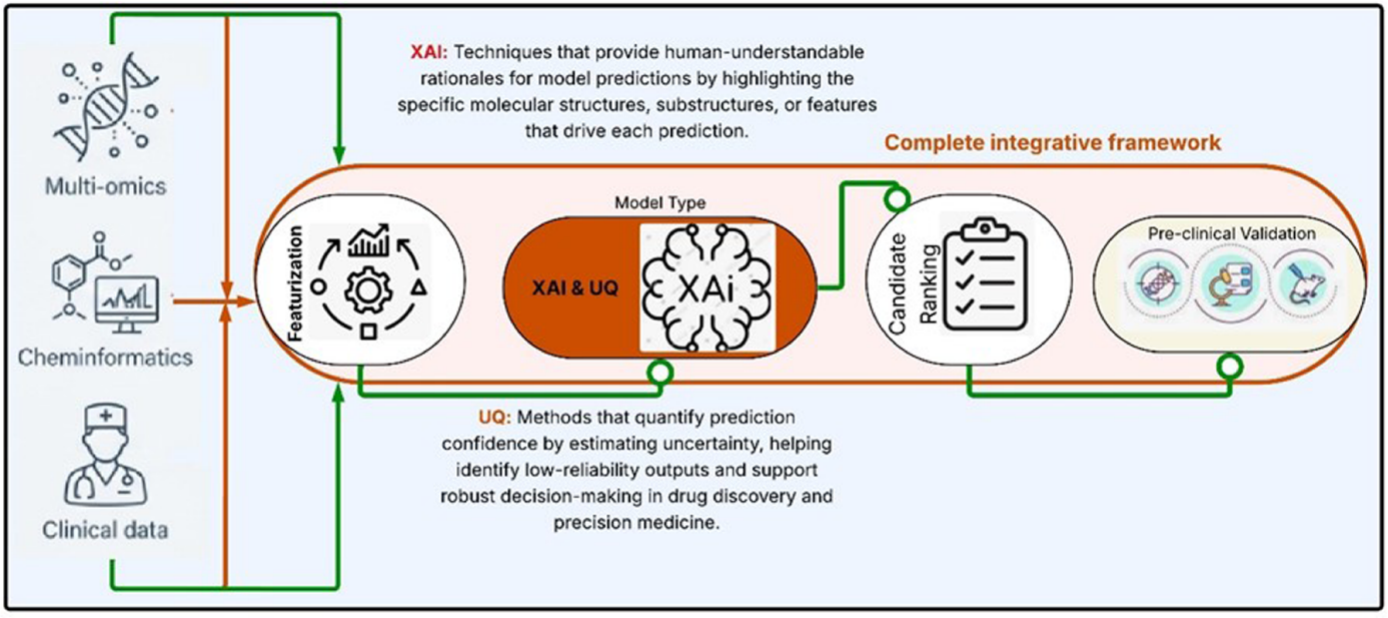
Integrative framework illustrating the use of multiomics, cheminformatics, and clinical data resources for XAI-based discovery of dual PPARα/γ agonists.

A robust AI pipeline should (i) inventory and link raw sources including AMP-AD, ADNI, ROSMAP, ChEMBL, BindingDB, PDB, and PRIDE; (ii) apply consistent QC and batch correction with version-controlled tools; (iii) reserve external validation cohorts; (iv) compute and report feature attributions (SHAP, Integrated Gradients) for top predictions; (v) quantify uncertainty and flag high-uncertainty candidates; (vi) produce a model card and deposit data under FAIR principles; and (vii) consider federated learning and secure aggregation for multi-institutional collaborations.

## 4 Digital Twin Frameworks for PPARα/γ Agonism in Alzheimer’s Disease

The concept of a digital twin a dynamic, computational replica of a biological system that evolves in parallel with its real-world counterpart has recently emerged as a powerful paradigm for precision medicine and systems pharmacology.^49^ In the context of AD, digital twins offer a unifying framework to integrate molecular, cellular, circuit-level, and clinical data into individualized, predictive models of disease progression and therapeutic response.^50^ For dual PPAR α/γ agonism, digital twins can be constructed by coupling multiomics profiles (genomics, transcriptomics, proteomics, metabolomics), neuroimaging biomarkers, and longitudinal clinical phenotypes with mechanistic models of PPAR-mediated metabolic and neuroimmune regulation.^51^ Such patient-specific or cohort-level twins enable in silico experimentation to evaluate how modulation of PPAR α and γ pathways influences glucose utilization, lipid metabolism, mitochondrial function, microglial activation, and amyloid-β clearance over time. By simulating dose–response relationships, target engagement, and downstream pathway rewiring, digital twins provide a means to explore therapeutic hypotheses that are impractical or ethically challenging to test directly in humans.^52^ Importantly, this approach aligns with the multifactorial nature of AD, where heterogeneous metabolic and inflammatory trajectories limit the effectiveness of one-size-fits-all interventions. Eventually, digital twin frameworks currently lack prospective, end-to-end validation demonstrating improved efficiency or precision relative to standard workflows. Quantitative metrics such as time-to-hit, hit-rate enrichment, and responder prediction accuracy remain necessary to substantiate these claims, however, comprehensive evaluation of integrated pipelines, including uncertainty propagation and system-level calibration, remains an open challenge.

## 5 Case Studies

The case studies encompass a range of PPAR-targeting strategies, including single-receptor and dual-receptor agonists. These examples are intended to demonstrate the application of AI methodologies in drug discovery and optimization rather than to establish mechanism-specific conclusions for dual PPARα/γ modulation.

### 5.1 AI-Optimized Combination Therapy with PPAR Agonists

In AD pathology, PPARγ and PPARα have been found to be involved in multiple neuroprotective processes, such as the reduction of inflammation, a boost in insulin sensitivity, and the control of lipid metabolism.^53^ Although single PPAR agonists have shown promise in preclinical and early clinical studies, the complex and multifactorial nature of AD suggests that combination strategies may offer greater therapeutic benefit.^54^ In this context, AI-driven approaches provide a promising avenue for optimizing such combination therapies, particularly by enabling data-driven selection and personalization of PPAR-targeted interventions.ML methods can scan large sets of data including genomics, proteomics, and clinical end points to determine potentially synergistic pairs of PPAR agonists or PPAR agonists and other drugs targeting distinct features of AD pathology.^55^ For example, AI could estimate that the combination of a PPARγ agonist such as pioglitazone and a PPARα agonist would treat simultaneously both neuroinflammation and lipid dysregulation in AD. In addition, AI may propose ideal combination dosages, potentially achieving maximum therapeutic gain with minimal side effects, which is important considering the long-term treatment generally needed for AD.^56^

In this regard, it might be most promising for AI research that it could assist with personalized medicine in AD patient. Through combining patient data including genetic risks, biomarkers, and imaging data, AI algorithms could divide these patients into groups who would most likely respond well to specific combinations of PPAR agonists.^57^ This would likely prove highly influential in shaping the form of clinical trials that would likely be utilized. Furthermore, using AI research regarding molecular interactions, there would be potential within this that new PPAR agonists could be identified or that drugs that currently exist could potentially be used in combination with PPAR agonists in treating AD. This could likely help with drug development.^58^ Nevertheless, it should be kept in mind that though AI-optimized combination therapy using PPAR agonists is highly promising for the treatment of AD, there are considerable challenges involved. The multiplicity of AD pathology, the selective permeability of the blood-brain barrier, and the risk of long-term side effects of PPAR agonists are all challenges that need to be considered with caution.^59^ In addition, AI algorithm predictions will need to be rigorously tested by preclinical studies and clinical trials. In spite of these obstacles, the use of AI for PPAR agonist combination therapy optimization is a strong method that can result in more efficient treatments for AD and possibly enhance the lives of millions suffering from this devastating disease.^60^ Ultimately, this example shows how AI-based modeling confirms mechanistic rationale for dual PPAR modulation for AD by predicting synergistic benefits from mixture therapies. The use of AI for identifying and optimizing multitarget mixtures with PPARα and PPARγ agonists sets the therapeutic importance of dual targeting of neuroinflammation and metabolic pathology. These findings conform to broader patterns that dual agonist strategies, when combined with AI-based optimization, consistently improve cognitive function and biomarker profiles in AD models.

### 5.2 Pioglitazone Therapy Optimization through Predictive Modeling

Since inconsistent results of pioglitazone *(PPARγ agonist)* in AD were seen in previous clinical trials, the scientists hypothesized that the potency of pioglitazone may be patient-level characteristic-dependent.^61^ To test this hypothesis, a SVM model was constructed based on clinical and biomarker information from 320 AD patients who had prior pioglitazone treatment.^62,63^ The model characterized a specific subgroup of patients with insulin resistance, inflammatory biomarker signatures, and the APOE ε3 genotype as treatment responders. A follow-up precision trial with 85 patients, filtered based on these AI-generated criteria, showed a 42% cognitive improvement over placebo, dramatically exceeding the 12% average improvement seen in the larger AD population.^64^ Such results depict the revolutionary power of AI-directed stratification in maximizing the use of current therapies, validating the merits of precision medicine strategies in AD treatment (Figure 7). The above case illustrates how ML-based patient stratification based on clinical and biomarker information has the potential to meaningfully boost the effectiveness of pioglitazone, a PPARγ selective agonist. The cognitive improvements observed in AI-identified subgroups support the therapeutic premise that precision targeting of PPAR pathways can yield meaningful clinical benefits. These findings suggest that AI-guided personalization of PPAR agonist therapy may enhance treatment response while reinforcing the mechanistic role of modulating metabolic and inflammatory pathways in AD.

**Figure 7:**
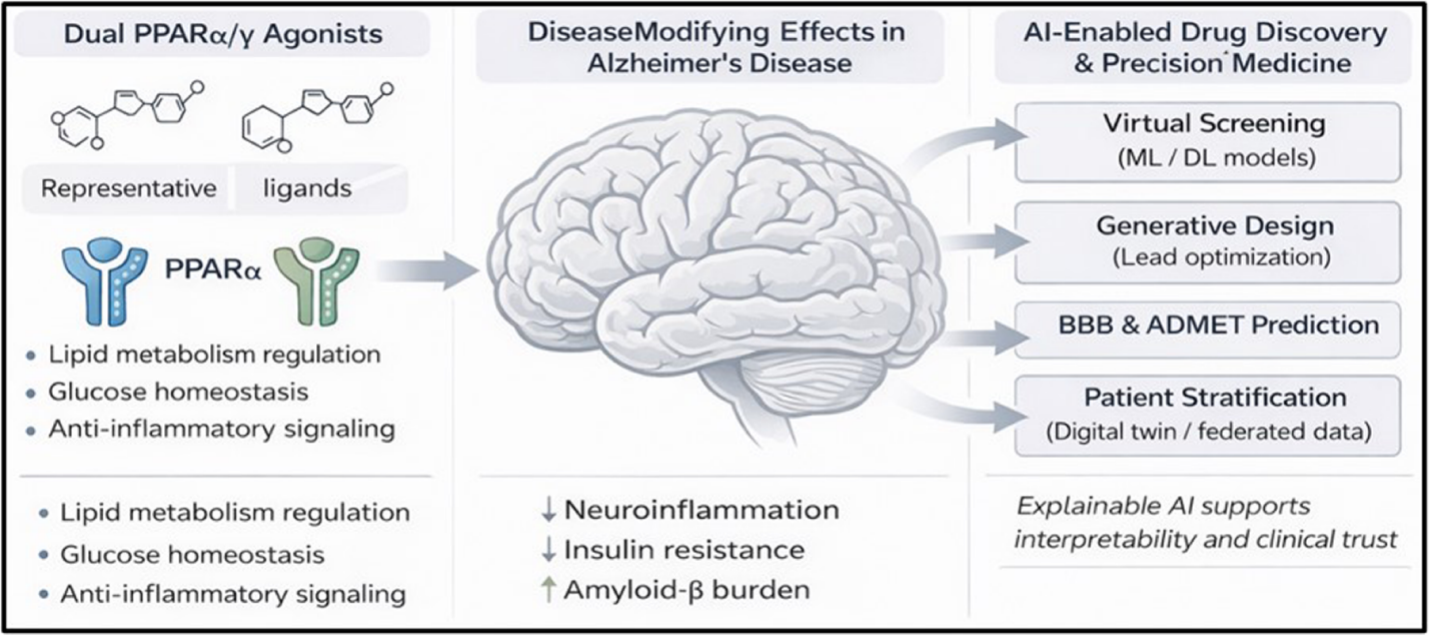
PPAR-targeted drugs for Alzheimer’s therapy: Key drug names, AD-relevant mechanisms, PPAR targets, development status, and applied AI/ML models for drug discovery and biomarker prediction. A conceptual overview and does not reflect quantitatively validated pathway modulation or comparative efficacy.

Moreover, a network-based AI platform to discover candidate targets for drug repurposing against AD. By integrating drug targets and Alzheimer’s-related genes (ARGs) into a brain-specific protein interaction network, the model pointed out pioglitazone’s promise to regulate PPARγ-mediated control of tauopathy-linked kinases, GSK3β and CDK5. Experimental verification in human microglia (HMC3 cells) validated that pioglitazone blocked LPS-induced phosphorylation of these kinases in a dose-dependent fashion, correlating AI predictions with concrete molecular effects pertinent to neurodegeneration.^65^

### 5.3 AI-Optimized Saroglitazar Dual PPARα/γ Therapy

The promise of saroglitazar (dual PPARα/γ agonist) used to treat dyslipidemia, in the therapy of early stage AD were investigated. A preclinical study proved that saroglitazar has neuroprotective properties in a scopolamine-induced rat model of AD, indicating its promise in reducing cognitive impairments of the disease.^66^ Also, deep learning has also proven promising in the prediction of the progression from MCI to AD. In recent study, an establishment of a deep RNN model that reliably predicts personalized disease trajectories, accentuating the value of such models in early intervention strategies.^67^ Lifestyle factors, specifically Mediterranean dietary habits and exercise, have been shown to lower risks of cognitive impairment and AD. In a systematic review and meta-analysis, higher adherence to the mediterranean diet was shown to lower risks of dementia and AD.^68^ Again, in a meta-analysis of prospective studies, physical activity was shown to lower risks of developing AD.^69^ A combination of pharmacologic therapies like saroglitazar, along with lifestyle modifications and the application of predictive models of AI, might hold the key to treating early stage AD patients specifically targeted to suit individual needs and characteristics. These multifactorial approaches are quite promising in deriving maximum benefit and postponing further degradation of disease conditions. The neuroprotective property of saroglitazar, as evident in deep learning-based predictive models, clearly established the importance of PPARα/γ dual agonism in AD modeling.^70^ In establishing AI-based prediction of disease progression and individualized interventions, this case presentation makes a significant case for addressing multiple disease issues, specifically metabolic and inflammatory diseases, simultaneously and together effectively (Table 5).

**Table 5:** Overview of Key Compounds Targeting PPAR Isoforms, Their Mechanisms Relevant to AD Pathology, Development Status, and Associated AI/ML Models Applied for Drug Discovery, Optimization, and Therapeutic Prediction

Drug Name	PPAR Target(s)	AD-Relevance Mechanism	Status	AI/ML Models Applied
Pioglitazone	PPAR-γ	Anti-inflammatory, Aβ clearance	Studied in trials	Gene expression clustering, ML-based patient stratification
Fenofibrate	PPAR-α	Lipid metabolism, oxidative stress	Preclinical	Virtual screening (QSAR, docking ML pipelines)
Saroglitazar	PPAR-α/γ (dual)	Neuroinflammation, metabolic regulation	Preclinical in AD	Deep learning for structure–activity prediction
Rosiglitazone	PPAR-γ	Cognitive effects, insulin signaling	Mixed trial results	Predictive toxicology (SVMs, ensemble classifiers)
GW7647	PPAR-α	Mitochondrial biogenesis	Experimental	Graph neural networks (GNNs) for binding prediction
T3D-959	PPAR-δ/γ (dual)	Brain insulin sensitivity	Phase II	AI-based pharmacokinetics simulation
Elafibranor	PPAR-α/δ (dual)	Metabolic-inflammation link	NASH studies	Target-ligand affinity prediction using CNNs
Bezafibrate	Pan (α/δ/γ)	Mitochondrial function	Neurodegeneration models	Omics-based biomarker prediction (Random Forest, XGBoost)

### 5.4 Lobeglitazone Combination Therapy Enhanced by Network Pharmacology

Recent studies develop an optimized combination therapy, insight driven, based on lobeglitazone (PPARγ agonist).^71^ In the study, the research group used knowledge GNN to analyze the complex disease networks in AD and identify the key nodes where intervention with lobeglitazone can be synergistically enhanced by other compounds.^72^ The AI model predicted that lobeglitazone administered together with a low-dosage BACE1 inhibitor and a special omega-3 fatty acid formulation would have synergistic effects on several AD pathways, such as amyloid clearance, neuroinflammation, and synaptic function.^73^ Experimental validation in transgenic AD mouse models confirmed these predictions, yielding superior efficacy over any single agent or random combination. A clinical trial involving 64 mild-to-moderate AD patients using the AI-based triple therapy regimen showed collective cognitive scores that were 31% better and amyloid levels measured by PET scans that were 28% lower at 12 months compared to the traditional approach.^65^ This case study not only demonstrates the application of AI in network analysis to rationally design combination therapies around PPAR agonists in AD but further proves that PPAR dual and selective modulation in AD is justified as it demonstrates that AI-driven combination therapies around lobeglitazone agonist and other complementary compounds do work in terms of enhancing cognitive and pathological efficacy in AD (Table 5). Likewise, network pharmacology analysis/GNNs not only reinforced traditional pathways but further developed new pathways by indicating that using AI in fine-tuning drug interactions is highly efficient in improving therapy in AD-related pathways by multiple mechanisms.

### 5.5 Elafibranor Precision Dosing Using Digital Biomarkers and Reinforcement Learning

Elafibranor (dual PPARα/δ agonist), initially used as a NASH treatment, was optimized using a precision dosing approach, specifically designed and tested for AD patients by Ratziu et al.^74^ and Westerouen et al.;^75^ a protocol further described by Levy et al.^76^ The researchers built a closed-loop platform that incorporated wearable devices, monitoring digital biomarkers such as sleep habits, physical activity, and heart rate variability, along with a dynamic dose-adjusting algorithm using a reinforcement learning algorithm that adjusted Elafibranor dosing.^75^ Elafibranor dosing was optimized by the AI, taking note of patient responses through their pharmacokinetic profiles. The AI platform led to a significant 28% enhancement and a 45% reduction in side effects compared to traditional dosing, as demonstrated by a group using AI-enhanced precision dosing compared to manual dosing among 78 moderately affected AD patients over a period of 9 months. The approach identified the best-possible therapeutic time windows for each patient and highlighted the important chronobiological aspects of the treatment’s effectiveness. Being the first case study, it illustrates the application of AI to make PPAR agonist treatment highly personal to each patient, as dictated by the specifics of the case in question. Moreover, the elafibranor approach to adjusting treatment according to the real-life, instantaneous physiological needs of the patient vindicates the application of AI in personalizing PPAR treatment modalities. Elafibranor, as a PPAR α/Δ*d*ual agonist, is proof of the concept of dual PPAR agonists being optimized to maximize effectiveness and safety via the application of reinforcement learning.

### 5.6 Novel Selective PPAR Modulator Developed through Generative AI

COMPASS-31, a selective PPAR modulator, was generated using generative AI. A very advanced form of GAN was used, where data was provided regarding known PPAR modulators, and their action on different physiological processes, which are related to AD.^77^ The aim of developing this AI was to generate compounds that preferentially activate those pathways of PPAR, which are beneficial in neuronal and glial cells, without affecting those pathways, which have negative consequences in peripheral tissues. Once generated, new compounds were synthesized and tested on animals.^78^ The lead compound, COMPASS-31, which emerged from this process, possessed some very special attributes, including acting as a partial PPARγ agonist, possessing tissue-selective profiles, and showing remarkable ability to cross the brain. In the transgenic AD mouse models, COMPASS-31 attenuated neuro-inflammatory markers by 67%, reduced the number of amyloid plaques by 43%, and enhanced cognitive function without the weight gain and edema seen in the traditional PPAR agonists. In early Phase 1 human studies, there was a positive safety profile, and Phase 2 studies regarding efficacy have initiated.^79^ This case study illustrates how AI-driven approaches can help overcome the traditional limitations of PPAR-targeted drugs by enabling the design of optimized candidates tailored to the pathophysiology of AD. Similarly, the development of COMPASS-31 using generative AI strategies highlights the potential of in silico drug discovery to address pharmacodynamic limitations associated with conventional PPAR agonists. The study confirms the applicability of AI in the rationale of targeted, PPAR pathway-specific modulation to produce improvements in the pathophysiological and cognitive realms of AD mouse models.

### 5.7 Integrative Multimodal Imaging and PPAR Activation Therapy

An intriguing case study was the work done by a research team from the Massachusetts General Hospital to develop an AI approach that interfaced with several neuroimaging techniques (MRI, fMRI, PET, and DTI) to personalize fenofibrate therapy in AD sufferers.^80^ This AI system analyzed the images to identify specific disease patterns in each patient based on vascular dysfunction, neuroinflammation, and connectivity abnormalities.^81,82^ Using these disease profiles, the AI system gave each patient specific suggestions to optimize fenofibrate therapy based on their own personal AD disease.^82^ However, since vascular disease was a major contributor to these AD sufferers’ intellectual deficits, the AI system suggested increased dosages of the drug as well as other treatments to aid vascular function. With a patient population of 103, this therapy approach with fenofibrate significantly increased blood flow to the cerebrum and corresponded with a stabilization of intellectual function in AD sufferers.^82^ Additionally, the personalized approach achieved 47% higher responder rate compared to standard protocols. This case study validates how AI analysis of complex neuroimaging data can guide the targeted application of PPAR agonist therapy to address patient-specific pathophysiological features of AD, moving beyond one-size-fits-all treatment approaches. This case confirms that integrating AI-analyzed neuroimaging with targeted PPARα therapy results in measurable improvements in cerebrovascular and cognitive function. The approach validates the dual-action model, targeting inflammation and metabolism, by aligning neuroimaging biomarkers with patient-specific therapeutic interventions. It supports the broader observation that AI-enhanced PPAR targeting, even with monotherapy, yields improved outcomes across diverse AD phenotypes.

For drugs like pioglitazone, ML models such as SVMs and decision trees have been employed to identify gene expression signatures and stratify patients based on likely response to anti-inflammatory treatment.^83^ Fenofibrate has been studied using QSAR-based virtual screening and machine learning–guided docking approaches to better understand its binding affinity and potential role in modulating oxidative stress.^23^ In the case of saroglitazar, deep learning algorithms have been applied to predict blood-brain barrier permeability and optimize its dual agonist properties for enhanced neuroprotective action.^84^ Bezafibrate, a pan-PPAR agonist, has been shown to improve mitochondrial function and reduce oxidative stress in neuronal systems. It has also been investigated using omics-integrated machine learning approaches, including random forest and XGBoost, to identify relevant gene targets.^85^ Elafibranor, a dual PPARα/δ agonist originally developed for NASH, may offer indirect neuroprotective benefits through systemic lipid and inflammation modulation, and has been evaluated using CNN-based prediction models for target engagement.^86,87^

### 5.8 AI Modeling of Metabolic Stress and PPAR Pathways

Metabolic dysregulation with a special focus on lipid homeostasis and mitochondrial function is being more and more identified as a central characteristic in AD pathogenesis.^88^ A thrilling addition to a model exists where, CNNs or graph-based models to process imaging inputs, while clinical and metabolic information are processed through structured input pipelines, all coming together in the form of attention layers to improve model interpretability and performance. Moreover, model performs directly incorporating metabolic biomarkers that are affected by PPAR agonists, for example, HDL, triglycerides, fatty acid oxidation indicators, or mitochondrial stress markers, into these AI models.^89^ In this way, scientists will be able to predict the way metabolic therapy, involving PPAR manipulation, will affect the process of neurodegeneration in an individualized manner. For instance, positive results shown by a PET scan in terms of increased brain glucose metabolism along with decreased systemic oxidative stress can be used to update these models. Such combined approaches can potentially not only help predict response to therapy but also select optimal patients for PPAR manipulation.^90^ Such predictive modeling with AI can transform precision neurology by coordinating metabolic therapy tactics with personal disease dynamics.^8^ A conceptual model for this framework would feature a multibranch model design in which imaging and omics data are fed into modality-specific encoders and then combined through attention or fusion layers to predict outcomes like cognitive decline or conversion of MCI to AD. Additionally, recent works integrate AI-based multimodal learning (neuroimaging + clinical/metabolic data) to predict disease progression.^91^ An extension might add metabolic biomarkers (such as lipid profiles to PPAR agonists) into AI models as predictors of neurodegeneration trajectories.

### 5.9 AI Model Predicting PPAR Binding Affinity for Drug Discovery

A regression model of DL, which was trained on more than 3,700 known small molecules with PPAR binding affinities. They were represented by 2D molecular descriptors and augmented with AutoDockVina docking scores to mimic receptor–ligand interactions. The output neural network displayed robust predictive power (R^2^ = 0.861 training, 0.655 test), suggesting that the model is worthy of prioritizing in silico high-affinity PPAR agonists.^92^ This method greatly decreases the cost and time of conventional high-throughput screening, enabling researchers to discover candidates that can modulate PPAR-α or PPAR-γ activity, both of which have been implicated in modulating lipid metabolism, mitochondrial function, and anti-inflammatory pathways involved in neurodegenerative disease.

While these studies collectively highlight the potential of AI-driven approaches, they involve heterogeneous pharmacological targets. Therefore, caution is required in attributing observed therapeutic effects specifically to dual PPARα/γ modulation without stratified comparative analyses.

## 6 Limitations

The bibliometric analysis presented in this study is based on co-occurrence network visualization and does not include statistical benchmarking such as null-model comparisons, enrichment analysis, temporal modularity assessment, or cross-citation validation. Therefore, the observed associations represent qualitative patterns of comention and should not be interpreted as definitive evidence of intellectual or collaborative integration between research domains. In addition, with, another key limitation, arises from the heterogeneity of included studies, encompassing diverse end points such as cognitive scales, imaging biomarkers, and molecular signatures. This variability limits direct comparability and precludes quantitative synthesis. Differences in study design and validation strategies may influence reported outcomes. Therefore, conclusions drawn from cross-study comparisons should be interpreted cautiously. Importantly, the inclusion of studies involving heterogeneous PPAR-targeting strategies, including single-receptor and alternative isoform agonists. While these studies provide valuable insights into AI-driven drug discovery approaches, they do not uniformly support mechanism-specific conclusions related to dual PPARα/γ modulation. Future studies incorporating stratified and comparative analyses are required to delineate the specific contributions of dual receptor activation. Moreover, a key limitation is the lack of quantitative, comparative studies evaluating dual PPARα/γ agonism against single-receptor activation across multiple biological end points. As a result, the concept of balanced modulation remains largely inferential and requires validation through dose–response and time-resolved experimental studies.

In the face of promising advances in AI/ML-based approaches for PPAR agonist-mediated treatment of AD, several key caveats remain to be acknowledged. The pathophysiological heterogeneity of AD is a major concern, as cohorts of patients that develop models may not adequately reflect the diverse manifestations of the disease. Most studies are based on data sets that reflect bias in demographic representation; however, ethnic minorities, females, and comorbidities that may limit the generalization of AI-based insights are significantly underrepresented.^78,93^ Importantly, the quality and richness of training data continue to pose a challenge, whereby most data sets lack longitudinal data or standardized biomarker measurements at multiple time points to date, limiting models in effectively predicting the course of disease or outcomes from treatments.^94^ The “black box” nature of many advanced deep learning methods remains a significant challenge, as regulatory bodies and clinicians are often reluctant to rely on treatment decisions derived from models whose reasoning is not fully transparent or verifiable. This interpretability problem is especially acute with more intricate neural network models applied to drug discovery and optimization. Furthermore, the extrapolation from in silico predictions to clinical efficacy is beset with major challenges, as illustrated by numerous AI-designed PPAR agonists with favorable computational profiles that failed to show efficacy in follow-up clinical trials. The blood-brain barrier continues to represent a persistent challenge, with most AI platforms struggling to predict CNS penetration of new compounds. Methodological challenges also remain, such as overfitting of models trained on small data sets, varying validation methods between studies, and challenges in combining multimodal data types (genomic, proteomic, clinical, and imaging) into unified analytical platforms. Lastly, the time-consuming and resource-heavy nature of creating and deploying advanced AI systems poses practical entry barriers to its general adoption, especially in resource-constrained settings where AD burden is exponentially growing.

## 7 Future Scenario

The future of AI/ML applications in the AD treatment with PPAR agonists is extremely optimiztic, with several future directions poised to break new ground. Overall, combined learning approaches provide a new paradigm that enables modern AI models to learn from decentralized data which distributed across many institutions without compromising data privacy, thereby providing more access to heterogeneous data. In addition, XAI models, such as those employing attention mechanisms and feature attribution methods, are being designed to remove the “black box” problem, providing transparent reasons for model predictions.^95,96^ Although multimodal data fusion through high-level fusion architectures will support more precise disease modeling, with future systems capable of weighing genomic, proteomic, metabolomic, neuroimaging, and digital biomarker data to effectively capture the depth and complexity of AD and PPAR-mediated intervention.

Moreover, reinforcement learning methods have great promise to customize treatment protocols, with increasingly refined capacity to learn therapeutic parameters that can update in a continuous manner to adjust to specific patient responses over time.^97^ Further, Quantum computing innovations will soon more than likely unleash unprecedented computing power for simulations of protein–ligand interactions and molecular modeling able to revolutionize the design of highly selective PPAR modulators with enhanced penetration through the blood-brain barrier and fewer off-target effects.^98^ Active learning frameworks will minimize trial design by efficiently enrolling patients most likely to offer informative data, decreasing sample size requirements and accelerating therapeutic development. The coming together of AI with other next-generation technologies, such as highly advanced brain-computer interfaces, extremely minimally invasive biosensors, and single-cell sequencing, will provide for unmatched monitoring of therapeutic outcomes at the cell level, which will allow AI systems to continuously iteratively improve upon therapeutic methods.^58^ Very early on, a suggested 3D-CNN-VideoSwinFormer model shows strong accuracy and AUC in classifying AD from cognitively normal subjects through single 3D MRI scans.^99^ This DL method provides an encouraging avenue for early, noninvasive AD diagnosis. Lastly, the creation of standalone AI platforms with self-directed capabilities to test and construct hypotheses for PPAR-mediated AD processes could decrease discovery timelines substantially, potentially uncovering new therapeutic paradigms, maybe not even imagined by human researchers. With the development of these technologies and regulatory frameworks adjusting to permit AI-facilitated medical innovation, we foresee a revolution in the detection, optimization, and deployment of PPAR-targeted therapeutics to precision treat AD with improved outcomes for millions of individuals globally.

### 7.1 Multidisciplinary Framework for Accelerated Discovery

In addition, the future of AD management via dual PPAR α/γ agonists is poised to undergo a paradigm shift through an Accelerated Discovery Cycle that integrates multilayered artificial intelligence frameworks, spanning generative, agentic, and XAI, to compress the translational timeline from molecule to medicine (Figure 8). This cycle begins with united learning, enabling the secure aggregation of geographically distributed, multimodal data sets encompassing patient genomics, transcriptomics, proteomics, neuroimaging, and real-world clinical records to train highly generalizable foundation models while preserving patient privacy and regulatory compliance. Such decentralized learning architectures are particularly critical for AD, where heterogeneous disease progression and population-specific genetic risk factors often limit the reproducibility of conventional models.^100,101^

**Figure 8:**
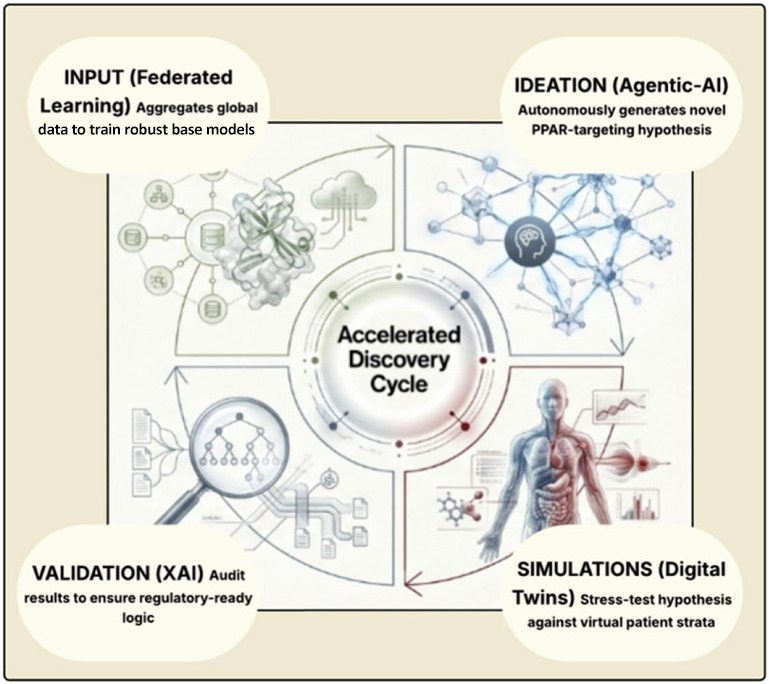
Illustration of integrated AI framework for accelerated PPARα/γ drug discovery in AD. Federated learning aggregates distributed biomedical data to train robust base models, agentic AI generates PPAR-targeting hypotheses, digital twin simulations evaluate therapeutic responses across virtual patient strata, and XAI ensures interpretability and regulatory readiness, collectively enabling a closed-loop accelerated discovery cycle for precision PPARα/γ therapeutics in Alzheimer’s disease.

Building upon these foundation models, Generative AI and Agentic-AI systems assume a proactive role in the ideation and optimization phases of drug discovery. Generative models explore vast chemical spaces to design novel dual PPAR α/γ agonists with optimized binding profiles, pharmacokinetics, and blood–brain barrier permeability.^38^ In parallel, Agentic-AI operates as an autonomous scientific collaborator, iteratively formulating hypotheses, prioritizing molecular candidates, orchestrating in silico screening campaigns, and adapting strategies based on feedback from simulation and experimental data.^102^

Candidate molecules generated through this agent-driven pipeline are subsequently evaluated using Digital Twin frameworks, which construct high-fidelity virtual representations of patient subpopulations.^103,104^ These digital twins integrate molecular, cellular, and systems-level data to simulate drug-target interactions, disease trajectories, and adverse effect profiles across genetically and metabolically diverse cohorts. By enabling in silico stratification of responders and nonresponders, this approach supports precision dosing strategies and minimizes late-stage clinical attrition, particularly in populations where dual PPAR modulation is predicted to confer maximal neuroprotective and disease-modifying benefits.^105^ Finally, to satisfy clinical translation and regulatory expectations, XAI is embedded throughout the discovery pipeline to ensure interpretability, traceability, and trustworthiness of model decisions. XAI frameworks elucidate the molecular determinants driving dual PPAR α/γ activation, clarify structure activity relationships, and expose causal links between predicted outcomes and underlying biological mechanisms. This transparency not only enhances scientific confidence but also facilitates regulatory review, ethical oversight, and clinician adoption. The convergence of Federated Learning, Generative-AI, Agentic-AI, Digital Twins, and XAI establishes a scalable, transparent, and patient-centric ecosystem for precision neurotherapeutics, positioning dual PPAR α/γ agonists as a cornerstone of next-generation AD treatment.

### 7.2 Validation, Generalization, and Benchmarking Considerations in AI-Driven PPAR Drug Discovery

While the progress of AI in drug discovery is fast paced, some methodological factors need to be considered to ensure that the predictions made through AI are robust and relevant, especially when developing dual PPARα/γ agonists for AD. Among these factors, the generalization ability of the predictive model is key. Most research tends to utilize internal validation methods like cross-validation. While effective at ensuring consistency within the model, such methods do not necessarily provide an accurate representation of the performance of the model on chemically different or unseen data.^106^ For CNS drug discovery, considering its unique challenges compared to most other cases, including physicochemical restrictions, permeability of the blood-brain barrier, and pharmacokinetic considerations, external validation is necessary.^107^

At the same time, comparing results with well-known approaches becomes essential to evaluate any additional value created by AI techniques. Traditional methods like molecular docking, classical QSAR analysis, and expert heuristics are popular in preclinical drug design.^108^ It is important to compare outcomes to these baseline techniques since only then one can understand whether AI-based prioritization creates any benefits.

One other equally critical factor is the difference between workflow concepts versus validated systems. Although integrated AI workflows combining generative models, multiomics data, explainable AI, and digital twins hold many potential benefits, actual validation using biochemical, cell-based, and in vivo tests is still scarce.^109^ Performance indicators such as improvements in hit rate enrichment, effect size, and time to lead are needed to back up any claim of rapid and precise discoveries. In addition, the relationship between AI-driven drug prioritization and physiological effects, such as neuroinflammation, glucose metabolism, and amyloid-β modulation should be viewed cautiously. The association is usually based on theoretical models that have not been experimentally verified. These factors highlight the necessity of incorporating external validation, benchmarking, uncertainty quantification, and experimental verification within the AI-enabled drug development process. It is imperative that these issues be addressed in order to leverage the potential of computational techniques to develop treatment options for dual PPARα/γ activation in AD. Eventually, systems-level integration of several AI technologies in closed loop drug discovery pipelines presents its own complexities, such as error propagation through different modules and difficulties in attributing performance improvements. At present, no prospective studies or evaluations have conclusively proved that such approaches result in superior results compared to traditional methods of drug discovery. In the absence of metrics such as lead time improvement, success probability improvement, or responder enrichment, quantification of improvements due to integration is hard to achieve.

## 8 Conclusions

AI combined with PPAR-targeted therapeutic strategies in such a manner represents a pivotal development for neuroimmune pharmacology in AD. AI-catalyzed discovery and optimization of dual PPARα/γ agonists provide a mechanistically grounded approach toward responding to the multifactorial nature of AD, simultaneously mitigating neuroinflammation, reducing metabolic homeostasis, and abating amyloid-β pathology. In tandem, ML, generative and agentic-AI systems, and XAI can be combined to enable the efficient multitarget ligand design, interpretable drug-receptor modeling, and rational optimization of pharmacokinetic and safety profiles. In parallel, federated learning frameworks shall support the privacy-preserving integration of large-scale multiomics and clinical data sets; digital twin paradigms enable in silico patient stratification while foretelling the therapeutic response, safety, and dosing before clinical assessment. The AI-enabled strategies establish a coherent translational pathway from molecular discovery to precision medicine, positioning dual PPARα/γ modulation as a promising and data-driven avenue to disease-modifying intervention in AD. These approaches offer promising frameworks for accelerating and refining drug discovery. However, their impact on efficiency and precision requires validation through systematic benchmarking and prospective evaluation. Future work integrating systems biology modeling and quantitative comparative analysis will be essential to validate the mechanistic advantages of dual PPARα/γ agonism.

## Notes

The authors did not use generative AI or AI assisted technologies in the writing of this manuscript.

## References

[1] 2025 Alzheimer’s disease facts and figures Alzheimers Dement. 2025, 21, e70235. 10.1002/alz.70235.

[2] Ardanaz C.
G., Ramirez M. J., Solas M. (2022). Brain Metabolic Alterations in Alzheimer’s
Disease. Int. J. Mol. Sci..

[3] Steinke I., Singh M., Amin R. (2024). Dual PPAR delta/gamma agonists offer
therapeutic potential for Alzheimer’s disease. Neural Regener. Res..

[4] Joardar A., Menzl J., Podolsky T. C., Manzo E., Estes P. S., Ashford S., Zarnescu D. C. (2015). PPAR gamma
activation is neuroprotective
in a Drosophila model of ALS based on TDP-43. Hum. Mol. Genet..

[5] Kariharan T., Nanayakkara G., Parameshwaran K., Bagasrawala I., Ahuja M., Abdel-Rahman E., Amin A. T., Dhanasekaran M., Suppiramaniam V., Amin R. H. (2015). Central activation of PPAR-gamma
ameliorates diabetes induced cognitive dysfunction and improves BDNF
expression. Neurobiol. Aging.

[6] Martinez A. A., Morgese M. G., Pisanu A., Macheda T., Paquette M. A., Seillier A., Cassano T., Carta A. R., Giuffrida A. (2015). Activation
of PPAR gamma receptors reduces levodopa-induced dyskinesias in 6-OHDA-lesioned
rats. Neurobiol. Dis..

[7] Priya D., Hani U., Haider N., Talath S., Shanmugarajan D., Prabitha P., Archana P., Kumar B. R. P. (2024). Novel PPAR-gamma
agonists as potential neuroprotective agents against Alzheimer’s
disease: rational design, synthesis, in silico evaluation, PPAR-gamma
binding assay and transactivation and expression studies. RSC Adv..

[8] Zulinska S., Strosznajder A. K., Strosznajder J. B. (2024). Current View on PPAR-alpha and Its
Relation to Neurosteroids in Alzheimer’s Disease and Other
Neuropsychiatric Disorders: Promising Targets in a Therapeutic Strategy. Int. J. Mol. Sci..

[9] Krishna S., Cheng B., Sharma D. R., Yadav S., Stempinski E. S., Mamtani S., Shah E., Deo A., Acherjee T., Thomas T. (2021). PPAR-gamma activation enhances myelination and neurological
recovery in premature rabbits with intraventricular hemorrhage. Proc. Natl. Acad. Sci. U. S. A..

[10] Latifi-Navid H., Mokhtari S., Taghizadeh S., Moradi F., Poostforoush-Fard D., Alijanpour S., Aghanoori M.-R. (2025). AI-assisted multi-OMICS analysis
reveals new markers for the prediction of AD. Biochim. Biophys. Acta, Mol. Basis Dis..

[11] Sarkar C., Das B., Rawat V. S., Wahlang J. B., Nongpiur A., Tiewsoh I., Lyngdoh N. M., Das D., Bidarolli M., Sony H. T. (2023). Artificial Intelligence and Machine
Learning Technology
Driven Modern Drug Discovery and Development. Int. J. Mol. Sci..

[12] Zhang Y., Yu L., Lv Y., Yang T., Guo Q. (2025). Artificial intelligence
in neurodegenerative diseases research: a bibliometric analysis since
2000. Front. Neurol..

[13] Huang J., Wang S., Liao X., Su D., Lin R., Zhang T., Zhao L. (2025). Knowledge map of artificial intelligence
in neurodegenerative diseases: a decade-long bibliometric and visualization
study. Front. Aging Neurosci..

[14] Amber-Vitos O., Chaturvedi N., Nachliel E., Gutman M., Tsfadia Y. (2016). The effect
of regulating molecules on the structure of the PPAR-RXR complex. Biochim. Biophys. Acta.

[15] Zhang B., Zhao J., Wang Z., Guo P., Liu A., Du G. (2021). Identification of Multi-Target Anti-AD Chemical Constituents
From
Traditional Chinese Medicine Formulae by Integrating Virtual Screening
and In Vitro Validation. Front. Pharmacol..

[16] Orasanu G., Ziouzenkova O., Devchand P. R., Nehra V., Hamdy O., Horton E. S., Plutzky J. (2008). The peroxisome proliferator-activated
receptor-gamma agonist pioglitazone represses inflammation in a peroxisome
proliferator-activated receptor-alpha-dependent manner in vitro and
in vivo in mice. J. Am. Coll Cardiol..

[17] Yoo J., Jeong I. K., Ahn K. J., Chung H. Y., Hwang Y. C. (2021). Fenofibrate,
a PPARalpha agonist, reduces hepatic fat accumulation through the
upregulation of TFEB-mediated lipophagy. Metabolism.

[18] Ezhilarasan D. (2024). Deciphering
the molecular pathways of saroglitazar: A dual PPAR alpha/gamma agonist
for managing metabolic NAFLD. Metabolism.

[19] Zhang W., Xiao D., Mao Q., Xia H. (2023). Role of neuroinflammation
in neurodegeneration development. Signal Transduction
Targeted Ther..

[20] Tong B., Ba Y., Li Z., Yang C., Su K., Qi H., Zhang D., Liu X., Wu Y., Chen Y., Ling J., Zhang J., Yin X., Yu P. (2024). Targeting
dysregulated lipid metabolism for the treatment of Alzheimer’s
disease and Parkinson’s disease: Current advancements and future
prospects. Neurobiol. Dis..

[21] Lee T.-W., Bai K.-J., Lee T.-I., Chao T.-F., Kao Y.-H., Chen Y.-J. (2017). PPARs modulate cardiac metabolism
and mitochondrial
function in diabetes. J. Biomed. Sci..

[22] Qie J., Liu Y., Wang Y., Zhang F., Qin Z., Tian S., Liu M., Li K., Shi W., Song L. (2022). Integrated
proteomic and transcriptomic landscape of macrophages in mouse tissues. Nat. Commun..

[23] Carpenter K. A., Huang X. (2018). Machine Learning-based
Virtual Screening and Its Applications to
Alzheimer’s Drug Discovery: A Review. Curr. Pharm. Des..

[24] Dandibhotla S., Samudrala M., Kaneriya A., Dakshanamurthy S. (2025). GNNSeq: A
Sequence-Based Graph Neural Network for Predicting Protein-Ligand
Binding Affinity. Pharmaceuticals.

[25] Qiu S., Cai Y., Yao H., Lin C., Xie Y., Tang S., Zhang A. (2023). Small molecule metabolites: discovery of biomarkers and therapeutic
targets. Signal Transduction Targeted Ther..

[26] Yang J., Sui H., Jiao R., Zhang M., Zhao X., Wang L., Deng W., Liu X. (2022). Random-Forest-Algorithm-Based Applications
of the Basic Characteristics and Serum and Imaging Biomarkers to Diagnose
Mild Cognitive Impairment. Curr. Alzheimer Res..

[27] Ren S., Lu Y., Zhang G., Xie K., Chen D., Cai X., Ye M. (2024). Integration of Graph Neural Networks and multi-omics analysis identify
the predictive factor and key gene for immunotherapy response and
prognosis of bladder cancer. J. Transl. Med..

[28] Tripathy, R. K. ; Frohock, Z. ; Wang, H. ; Cary, G. A. ; Keegan, S. ; Carter, G. W. ; Li, Y. An explainable graph neural network approach for effectively integrating multi-omics with prior knowledge to identify biomarkers from interacting biological domains bioRxiv 2024 10.1101/2024.08.23.609465

[29] Yoon J. T., Lee K. M., Oh J. H., Kim H. G., Jeong J. W. (2024). Insights
and Considerations in Development and Performance Evaluation of Generative
Adversarial Networks (GANs): What Radiologists Need to Know. Diagnostics.

[30] Sivarajkumar S., Gao F., Denny P., Aldhahwani B., Visweswaran S., Bove A., Wang Y. (2024). Mining Clinical
Notes for Physical
Rehabilitation Exercise Information: Natural Language Processing Algorithm
Development and Validation Study. JMIR Med.
Inform..

[31] Shankar R., Bundele A., Mukhopadhyay A. (2025). Natural language
processing of electronic
health records for early detection of cognitive decline: a systematic
review. NPJ. Digit. Med..

[32] Han L., Shen W. J., Bittner S., Kraemer F. B., Azhar S. (2017). PPARs: regulators
of metabolism and as therapeutic targets in cardiovascular disease.
Part II: PPAR-beta/delta and PPAR-gamma. Future
Cardiol..

[33] Vaz J. M., Balaji S. (2021). Convolutional neural networks (CNNs): concepts and
applications in pharmacogenomics. Mol. Diversity.

[34] Sharma A., Lysenko A., Jia S., Boroevich K. A., Tsunoda T. (2024). Advances in AI and machine learning for predictive
medicine. J. Hum. Genet..

[35] Liu Y.-T., Zhang L.-L., Jiang Z.-Y., Tian X.-S., Li P.- L., Wu P.-H., Du W.-T., Yuan B.-Y., Xie C., Bu G.-L. (2020). Applications
of Artificial Intelligence in Biotech
Drug Discovery and Product Development. MedComm.

[36] Shahid S. B., Kaikaus M., Kabir M. H., Yousuf M. A., Azad A. K. M., Al-Moisheer A. S., Alotaibi N., Alyami S. A., Bhuiyan T., Moni M. A. (2025). Novel deep learning for multi-class
classification of Alzheimer’s in disability using MRI datasets. Front. Bioinform..

[37] Gangwal A., Ansari A., Ahmad I., Azad A. K., Kumarasamy V., Subramaniyan V., Wong L. S. (2024). Generative artificial intelligence
in drug discovery: basic framework, recent advances, challenges, and
opportunities. Front. Pharmacol..

[38] Khater T., Alkhatib S. A., AlShehhi A., Pitsalidis C., Pappa A. M., Ngo S. T., Chan V., Truong V. K. (2025). Generative
artificial intelligence based models optimization towards molecule
design enhancement. J. Cheminform..

[39] Kang S. I., Shin J. H., Wu B. M., Choi H. S. (2025). Deep Generative
AI for Multi-Target Therapeutic Design: Toward Self-Improving Drug
Discovery Framework. Int. J. Mol. Sci..

[40] Liawrungrueang W. (2025). Artificial
Intelligence (AI) Agents Versus Agentic AI: What’s the Effect
in Spine Surgery?. Neurospine.

[41] Ekambaram S., Dokholyan N. V. (2026). Peptide-based
drug design using generative AI. Chem. Commun..

[42] Veitch D.
P., Weiner M. W., Aisen P. S., Beckett L. A., DeCarli C., Green R. C., Harvey D., Jack C. R., Jagust W., Landau S. M., Morris J. C., Okonkwo O., Perrin R. J., Petersen R. C., Rivera-Mindt M., Saykin A. J., Shaw L. M., Toga A. W., Tosun D., Trojanowski J. Q. (2022). Using the
Alzheimer’s Disease Neuroimaging Initiative
to improve early detection, diagnosis, and treatment of Alzheimer’s
disease. Alzheimers Dement.

[43] Cheng F., Wang F., Tang J., Zhou Y., Fu Z., Zhang P., Haines J. L., Leverenz J. B., Gan L., Hu J., Rosen-Zvi M., Pieper A. A., Cummings J. (2024). Artificial intelligence
and open science in discovery of disease-modifying medicines for Alzheimer’s
disease. Cell Rep. Med..

[44] Perez-Riverol Y. (2022). Proteomic
repository data submission, dissemination, and reuse: key messages. Expert Rev. Proteomics.

[45] Perez-Gonzalez A. P., Garcia-Kroepfly A. L., Perez-Fuentes K. A., Garcia-Reyes R. I., Solis-Roldan F. F., Alba-Gonzalez J. A., Hernandez-Lemus E., de Anda-Jauregui G. (2024). The ROSMAP
project: aging and neurodegenerative diseases
through omic sciences. Front. Neuroinform..

[46] Carracedo-Reboredo P., Linares-Blanco J., Rodriguez-Fernandez N., Cedron F., Novoa F. J., Carballal A., Maojo V., Pazos A., Fernandez-Lozano C. (2021). A review on
machine learning approaches and trends in drug discovery. Comput. Struct. Biotechnol. J..

[47] Ferreira F. J. N., Carneiro A. S. (2025). AI-Driven Drug Discovery:
A Comprehensive Review. ACS Omega.

[48] Moassefi M., Singh Y., Conte G. M., Khosravi B., Rouzrokh P., Vahdati S., Safdar N., Moy L., Kitamura F., Gentili A., Lakhani P., Kottler N., Halabi S. S., Yacoub J. H., Hou Y., Younis K., Erickson B. J., Krupinski E., Faghani S. (2024). Checklist for Reproducibility of
Deep Learning in Medical Imaging. J. Imaging
Inform. Med..

[49] Saratkar S. Y., Langote M., Kumar P., Gote P., Weerarathna I. N., Mishra G. V. (2025). Digital twin for personalized medicine
development. Front. Digit. Health.

[50] Li X., Loscalzo J., Mahmud A. K. M. F., Aly D. M., Rzhetsky A., Zitnik M., Benson M. (2025). Digital twins as global learning
health and disease models for preventive and personalized medicine. Genome Med..

[51] Emmert-Streib F., Parkkila S., Laubenbacher R., Mannermaa A., Hood L., Yli-Harja O. (2025). The role of
digital twins in P4 medicine:
A paradigm for modern healthcare. NPJ. Digit
Med..

[52] Akbarialiabad H., Pasdar A., Murrell D. F., Mostafavi M., Shakil F., Safaee E., Leachman S. A., Haghighi A., Tarbox M., Bunick C. G., Grada A. (2025). Enhancing
randomized
clinical trials with digital twins. Npj Syst.
Biol. Appl..

[53] Heneka M. T., Carson M. J., El Khoury J., Landreth G. E., Brosseron F., Feinstein D. L., Jacobs A. H., Wyss-Coray T., Vitorica J., Ransohoff R. M. (2015). Neuroinflammation in
Alzheimer’s disease. Lancet Neurol..

[54] Cheng H., Shang Y., Jiang L., Shi T. L., Wang L. (2016). The peroxisome
proliferators activated receptor-gamma agonists as therapeutics for
the treatment of Alzheimer’s disease and mild-to-moderate Alzheimer’s
disease: a meta-analysis. Int. J. Neurosci..

[55] Pushpakom S., Iorio F., Eyers P. A., Escott K. J., Hopper S., Wells A., Doig A., Guilliams T., Latimer J., McNamee C., Norris A., Sanseau P., Cavalla D., Pirmohamed M. (2019). Drug repurposing: progress, challenges
and recommendations. Nat. Rev. Drug Discovery.

[56] Vamathevan J., Clark D., Czodrowski P., Dunham I., Ferran E., Lee G., Li B., Madabhushi A., Shah P., Spitzer M., Zhao S. (2019). Applications of machine learning in drug discovery and development. Nat. Rev. Drug Discovery.

[57] Hampel H., Vergallo A., Aguilar L. F., Benda N., Broich K., Cuello A. C., Cummings J., Dubois B., Federoff H. J., Fiandaca M., Genthon R., Haberkamp M., Karran E., Mapstone M., Perry G., Schneider L. S., Welikovitch L. A., Woodcock J., Baldacci F., Lista S. (2018). Precision
pharmacology for Alzheimer’s disease. Pharmacol. Res..

[58] Paul D., Sanap G., Shenoy S., Kalyane D., Kalia K., Tekade R. K. (2021). Artificial intelligence in drug discovery
and development. Drug Discov. Today.

[59] Cummings J., Lee G., Zhong K., Fonseca J., Taghva K. (2021). Alzheimer’s
disease drug development pipeline: 2021. Alzheimers
Dement.

[60] Qiu Y., Cheng F. (2024). Artificial intelligence
for drug discovery and development in Alzheimer’s
disease. Curr. Opin. Struct. Biol..

[61] Saunders A. M., Burns D. K., Gottschalk W. K. (2021). Reassessment
of Pioglitazone for
Alzheimer’s Disease. Front. Neurosci..

[62] De
Felice F. G., Ferreira S. T. (2014). Inflammation, defective insulin signaling,
and mitochondrial dysfunction as common molecular denominators connecting
type 2 diabetes to Alzheimer disease. Diabetes.

[63] De
Sousa R. A. L., Harmer A. R., Freitas D. A., Mendonca V. A., Lacerda A. C. R., Leite H. R. (2020). An update on potential links between
type 2 diabetes mellitus and Alzheimer’s disease. Mol. Biol. Rep..

[64] Heneka M. T., Fink A., Doblhammer G. (2015). Effect of
pioglitazone medication
on the incidence of dementia. Ann. Neurol..

[65] Fang J., Zhang P., Wang Q., Chiang C. W., Zhou Y., Hou Y., Xu J., Chen R., Zhang B., Lewis S. J. (2022). Artificial
intelligence framework identifies candidate targets for
drug repurposing in Alzheimer’s disease. Alzheimers Res. Ther..

[66] Sandeep
Ganesh G., Konduri P., Kolusu A. S., Namburi S. V., Chunduru B. T. C., Nemmani K. V. S., Samudrala P. K. (2023). Neuroprotective
Effect of Saroglitazar on Scopolamine-Induced Alzheimer’s in
Rats: Insights into the Underlying Mechanisms. ACS Chem. Neurosci..

[67] Jung W., Jun E., Suk H. I. (2021). Deep recurrent
model for individualized prediction
of Alzheimer’s disease progression. Neuroimage.

[68] Nucci D., Sommariva A., Degoni L. M., Gallo G., Mancarella M., Natarelli F., Savoia A., Catalini A., Ferranti R., Pregliasco F. E. (2024). Association between Mediterranean diet and
dementia and Alzheimer disease: a systematic review with meta-analysis. Aging Clin. Exp. Res..

[69] Zhang X., Li Q., Cong W., Mu S., Zhan R., Zhong S., Zhao M., Zhao C., Kang K., Zhou Z. (2023). Effect of
physical activity on risk of Alzheimer’s disease: A systematic
review and meta-analysis of twenty-nine prospective cohort studies. Ageing Res. Rev..

[70] Cummings J. L., Osse A. M. L., Kinney J. W., Cammann D., Chen J. (2024). Alzheimer’s
Disease: Combination Therapies and Clinical Trials for Combination
Therapy Development. CNS Drugs.

[71] Wang J., Liu X., Shen S., Deng L., Liu H. (2022). DeepDDS: deep graph
neural network with attention mechanism to predict synergistic drug
combinations. Brief Bioinform..

[72] Hu X., Sun Z., Nian Y., Wang Y., Dang Y., Li F., Feng J., Yu E., Tao C. (2024). Self-Explainable Graph
Neural Network for Alzheimer Disease and Related Dementias Risk Prediction:
Algorithm Development and Validation Study. JMIR Aging.

[73] Roney M., Uddin M. N., Khan A. A., Fatima S., Mohd Aluwi M. F. F., Hamim S. M. I., Ahmad A. (2025). Repurposing
of dipeptidyl peptidase
FDA-approved drugs in Alzheimer’s disease using network pharmacology
and in-silico approaches. Comput. Biol. Chem..

[74] Ratziu V., Harrison S. A., Francque S., Bedossa P., Lehert P., Serfaty L., Romero-Gomez M., Boursier J., Abdelmalek M., Caldwell S. (2016). Elafibranor,
an Agonist of the Peroxisome Proliferator-Activated
Receptor-alpha and -delta, Induces Resolution of Nonalcoholic Steatohepatitis
Without Fibrosis Worsening. Gastroenterology.

[75] Westerouen
Van Meeteren M. J., Drenth J. P. H., Tjwa E. (2020). Elafibranor: a potential
drug for the treatment of nonalcoholic steatohepatitis (NASH). Expert Opin. Invest. Drugs.

[76] Levy C., Abouda G. F., Bilir B. M., Bonder A., Bowlus C. L., Campos-Varela I., Cazzagon N., Chandok N., Cheent K., Cortez-Pinto H., Demir M., Dill M. T., Eksteen B., Fenkel J. M., Gilroy R., Ko H. H., Jacobson I. M., Kallis Y., Kugelmas M., Luketic V., Mangia A., Montano-Loza A. J., Mukhopadhya A., Olveira A., Patel B. C., Pietrangelo A., Pradhan F., Salcedo M., Shiffman M. L., Sprinzl K., Swann R., Thorburn D., Thuluvath P. J., Trivedi P. J., Turnes J., Zein C. O., Gomes
da Silva H., Jaitly S., Miller B., Milligan C., Tavenard A., Kowdley K. V. (2026). Safety and efficacy of elafibranor
in primary sclerosing cholangitis: The ELMWOOD phase II randomized-controlled
trial. J. Hepatol..

[77] Hilz M. J., Wang R., Singer W. (2022). Validation
of the Composite Autonomic
Symptom Score 31 in the German language. Neurol.
Sci..

[78] Chen J., Zhang Y., Wang J., Xia Y., Zhang L., Chen L. (2024). Recent advances in Alzheimer’s
disease: Mechanisms, clinical
trials and new drug development strategies. Signal Transduction Targeted Ther..

[79] D’Amato C., Greco C., Lombardo G., Frattina V., Campo M., Cefalo C. M. A., Izzo V., Lauro D., Spallone V. (2020). The diagnostic
usefulness of the combined COMPASS 31 questionnaire and electrochemical
skin conductance for diabetic cardiovascular autonomic neuropathy
and diabetic polyneuropathy. J. Peripher. Nerv.
Syst..

[80] Leming M., Das S., Im H. (2023). Adversarial confound regression and uncertainty measurements
to classify heterogeneous clinical MRI in Mass General Brigham. PLoS One.

[81] Demuth S., Paris J., Faddeenkov I., De Seze J., Gourraud P. A. (2025). Clinical
applications of deep learning in neuroinflammatory diseases: A scoping
review. Rev. Neurol..

[82] Tang A. S., Oskotsky T., Havaldar S., Mantyh W. G., Bicak M., Solsberg C. W., Woldemariam S., Zeng B., Hu Z., Oskotsky B. (2022). Deep phenotyping
of Alzheimer’s disease
leveraging electronic medical records identifies sex-specific clinical
associations. Nat. Commun..

[83] Park M.-K., Ahn J., Lim J.-M., Han M., Lee J.-W., Lee J.-C., Hwang S.-J., Kim K.-C. (2024). A Transcriptomics-Based
Machine Learning
Model Discriminating Mild Cognitive Impairment and the Prediction
of Conversion to Alzheimer’s Disease. Cells.

[84] Das S., Gupta S., Lathia T., Swain J., Mittal S., Kumar S., Dm M., Garg N., Teja R., Beatrice A. (2025). Evaluation
of Effectiveness and Tolerability
of Saroglitazar in Metabolic Disease Patients of India: A Retrospective,
Observational, Electronic Medical Record-Based Real-World Evidence
Study. Cureus.

[85] Durai P., Beeraka N. M., Ramachandrappa H. V.
P., Krishnan P., Gudur P., Raghavendra N. M., Ravanappa P. K. B. (2022). Advances
in PPARs Molecular Dynamics and Glitazones as a Repurposing Therapeutic
Strategy through Mitochondrial Redox Dynamics against Neurodegeneration. Curr. Neuropharmacol..

[86] Blair H. A. (2024). Elafibranor:
First Approval. Drugs.

[87] Xu X., Poulsen K. L., Wu L., Liu S., Miyata T., Song Q., Wei Q., Zhao C., Lin C., Yang J. (2022). Targeted therapeutics and novel signaling pathways
in non-alcohol-associated
fatty liver/steatohepatitis (NAFL/NASH). Signal
Transduction Targeted Ther..

[88] Li X., Wu Z., Si X., Li J., Wu G., Wang M. (2025). The role of
mitochondrial dysfunction in the pathogenesis of Alzheimer’s
disease and future strategies for targeted therapy. Eur. J. Med. Res..

[89] Yan X., Hu Y., Wang B., Wang S., Zhang X. (2020). Metabolic Dysregulation
Contributes to the Progression of Alzheimer’s Disease. Front. Neurosci..

[90] Cunnane S. C., Trushina E., Morland C., Prigione A., Casadesus G., Andrews Z. B., Beal M. F., Bergersen L. H., Brinton R. D., de la Monte S., Eckert A., Harvey J., Jeggo R., Jhamandas J. H., Kann O., la Cour C. M., Martin W. F., Mithieux G., Moreira P. I., Murphy M. P., Nave K. A., Nuriel T., Oliet S. H. R., Saudou F., Mattson M. P., Swerdlow R. H., Millan M. J. (2020). Brain energy rescue:
an emerging therapeutic concept for neurodegenerative disorders of
ageing. Nat. Rev. Drug Discovery.

[91] Maddury S., Desai K. (2023). DeepAD: A deep learning
application for predicting amyloid standardized
uptake value ratio through PET for Alzheimer’s prognosis. Front. Artif. Intell..

[92] Ode Aman, L. ; Asnawi, A. AI Model for Predicting Binding Affinity of Antidiabetic Compounds Targeting PPAR. arXiv 2024.

[93] Kamatham P. T., Shukla R., Khatri D. K., Vora L. K. (2024). Pathogenesis, diagnostics,
and therapeutics for Alzheimer’s disease: Breaking the memory
barrier. Ageing Res. Rev..

[94] Wang T., Qiu R. G., Yu M. (2018). Predictive Modeling
of the Progression
of Alzheimer’s Disease with Recurrent Neural Networks. Sci. Rep..

[95] Ali S., Akhlaq F., Imran A. S., Kastrati Z., Daudpota S. M., Moosa M. (2023). The enlightening role
of explainable artificial intelligence in medical
& healthcare domains: A systematic literature review. Comput. Biol. Med..

[96] Muhammad D., Bendechache M. (2024). Unveiling the black box: A systematic review of Explainable
Artificial Intelligence in medical image analysis. Comput. Struct. Biotechnol. J..

[97] Leony F., Lin C. J. (2025). Multimodal fusion
architectures for Alzheimer’s
disease diagnosis: An experimental study. J.
Biomed. Inform..

[98] Vatansever S., Schlessinger A., Wacker D., Kaniskan H. U., Jin J., Zhou M. M., Zhang B. (2021). Artificial intelligence and machine
learning-aided drug discovery in central nervous system diseases:
State-of-the-arts and future directions. Med.
Res. Rev..

[99] Zhou J., Wei Y., Li X., Zhou W., Tao R., Hua Y., Liu H. (2025). A deep learning model for early diagnosis of alzheimer’s disease
combined with 3D CNN and video Swin transformer. Sci. Rep..

[100] Meng X., Yan X., Zhang K., Liu D., Cui X., Yang Y., Zhang M., Cao C., Wang J., Wang X., Gao J., Wang Y. G., Ji J. M., Qiu Z., Li M., Qian C., Guo T., Ma S., Wang Z., Guo Z., Lei Y., Shao C., Wang W., Fan H., Tang Y. D. (2024). The application
of large language models in medicine: A scoping review. iScience.

[101] Tam T. Y. C., Sivarajkumar S., Kapoor S., Stolyar A. V., Polanska K., McCarthy K. R., Osterhoudt H., Wu X., Visweswaran S., Fu S. (2024). A framework for human
evaluation of large language models in healthcare derived from literature
review. NPJ. Digit Med..

[102] Tang X., Jin Q., Zhu K., Yuan T., Zhang Y., Zhou W., Qu M., Zhao Y., Tang J., Zhang Z. (2025). Risks of AI scientists:
prioritizing safeguarding over autonomy. Nat.
Commun..

[103] Vallee A. (2023). Digital twin for healthcare systems. Front Digit Health.

[104] Ma C., Zhang H., Rao Y., Jiang X., Liu B., Sun Z., Song Z., Gao Y., Cui Y., Liu X., Li Z. (2025). AI-driven virtual cell
models in preclinical research: technical
pathways, validation mechanisms, and clinical translation potential. NPJ Digit. Med..

[105] Dette C. M. U., Alberg V., Rudesheim S., Selzer D., Marok F. Z., Bragazzi N. L., Fuhr L. M., Brunak S., Pearson E. R., Zahn T., Kiritsi D., Schwab M., Lehr T. (2025). Advancing rare disease therapeutics
through digital twins: Opportunities in drug development and precision
dosing. Comput. Struct. Biotechnol. J..

[106] Blanco-González A., Cabezón A., Seco-González A., Conde-Torres D., Antelo-Riveiro P., Piñeiro Á., Garcia-Fandino R. (2023). The Role of
AI in Drug Discovery: challenges, Opportunities, and Strategies. Pharmaceuticals.

[107] Hammarlund-Udenaes M., Loryan I. (2025). Assessing central nervous
system
drug delivery. Expert Opin. Drug Deliv..

[108] Fu C., Chen Q. (2025). The future
of pharmaceuticals: Artificial intelligence
in drug discovery and development. J. Pharm.
Anal..

[109] Hsu C. Y., Askar S., Alshkarchy S. S., Nayak P. P., Attabi K. A. L., Khan M. A., Mayan J. A., Sharma M. K., Islomov S., Soleimani Samarkhazan H. (2026). AI-driven
multi-omics integration in precision oncology: bridging the data deluge
to clinical decisions. Clin. Exp. Med..

